# Metabolic Variation during Development in Culture of *Leishmania donovani* Promastigotes

**DOI:** 10.1371/journal.pntd.0001451

**Published:** 2011-12-20

**Authors:** Ana Marta Silva, Anabela Cordeiro-da-Silva, Graham H. Coombs

**Affiliations:** 1 Strathclyde Institute of Pharmacy and Biomedical Sciences, University of Strathclyde, Glasgow, United Kingdom; 2 Instituto de Biologia Molecular e Celular, Universidade do Porto, Porto, Portugal; 3 Laboratório de Ciências Biológicas, Faculdade de Farmácia da Universidade do Porto, Porto, Portugal; Institut Pasteur, France

## Abstract

The genome sequencing of several *Leishmania* species has provided immense amounts of data and allowed the prediction of the metabolic pathways potentially operating. Subsequent genetic and proteomic studies have identified stage-specific proteins and putative virulence factors but many aspects of the metabolic adaptations of *Leishmania* remain to be elucidated. In this study, we have used an untargeted metabolomics approach to analyze changes in the metabolite profile as promastigotes of *L. donovani* develop during *in vitro* cultures from logarithmic to stationary phase. The results show that the metabolomes of promastigotes on days 3–6 of culture differ significantly from each other, consistent with there being distinct developmental changes. Most notable were the structural changes in glycerophospholipids and increase in the abundance of sphingolipids and glycerolipids as cells progress from logarithmic to stationary phase.

## Author Summary

*Leishmania* infections are considered neglected tropical diseases as the parasites affect millions of people worldwide but there are limited research efforts aimed at obtaining vaccines and new drugs. *Leishmania* has a digenetic life cycle alternating between promastigote forms, which develop in the sand-fly, the vector of the disease, and an amastigote form, which grows in mammals after being bitten by an infected sand-fly. *In vitro* studies with the promastigote forms are routinely used to gain insights about the parasite's cell biology. Little is known about how the different promastigotes forms are metabolically adapted to their particular micro-environment in the host or how they are pre-adapted metabolically for infecting a mammal, thus we have undertaken a study of the metabolite profile of *L. donovani* promastigotes in order to gain an understanding of the changes that occur during promastigote development. The analysis has revealed that the changes in promastigotes' metabolome between days 3 and 6 take place in a progressive manner; however major differences were observed when comparing the promastigotes on days 3 and 6. An increase in lipid abundance as promastigote development occurred was notable and is likely to reflect remodelling of the parasite's surface in readiness for infecting a mammal.

## Introduction

Leishmaniasis remains one of the major infectious diseases with 350 million people at risk in 88 countries worldwide and 2 million estimated new cases every year [1]. The lack of effective chemotherapy and emergence of drug resistance (reviewed in [2]) highlights the need for an improved knowledge of the parasite's cell biology to discover peculiarities that could potentially be explored as drug targets.

The *Leishmania* life cycle involves several developmental stages and alternates between sand fly and mammalian hosts. A major developmental difference is the occurrence as intracellular amastigotes in mammalian macrophages and as extracellular promastigotes in the sand fly. However, multiple forms of promastigotes have been identified based on morphology, location, infectivity, growth rate, ability to divide, and specific features such as expression of surface molecules [3]–[8]. It is believed that the parasite's occurrence in different developmental forms is a mechanism whereby it adapts to survive and persist in the various environmental conditions in which it is confronted with variations in temperature, pH, nutrient and oxygen availability and exposure to reactive oxygen (ROS) and nitrogen species (RNS) [9]. Despite the extensive investigations on various features of *Leishmania* over many years and the recent pioneering application of metabolomics technologies to studies on the parasite [10]–[13], particularly the elucidation of ways in which amastigotes differ from promastigotes [13]–[16], currently relatively little is known about the detail of the metabolic variation that happens during this developmental sequence in the sand fly.

The developmental sequence in the sand fly vector, which terminates in transformation to the metacyclic form infective to a mammalian host, appears to be mimicked, at least in part, during growth axenically in vitro; this comprises multiplication of procyclic promastigote forms and then differentiation to the metacyclic form, a process known as metacyclogenesis which is accompanied by morphological changes, including reduction in size of the cell body and a relatively longer flagellum, and some known biochemical changes such as lipophosphoglycan (LPG) and surface protein expression [5]–[7], [17]–[20]. Thus the in vitro system provides an opportunity to investigate the metabolome changes that accompany and perhaps underpin the developmental sequence of the promastigote. In the present study, we have applied state-of-the-art metabolomics approaches to analyse the changes in the metabolome of promastigotes of *Leishmania donovani* during culture in vitro. The results show that there is distinct variation in the metabolome, especially in the lipid composition.

## Methods

### *Leishmania* parasites

*Leishmania donovani* (MHOM/NP/03/BPK206/0clone10) promastigotes had been cloned from an isolate from a visceral leishmaniasis patient sensitive to pentavalent antimonials in Nepal, as described by Rijal and co-workers [21]. Promastigotes were grown on modified Eagle's medium (designated HOMEM medium, Invitrogen) supplemented with 20% (v/v) heat inactivated fetal calf serum (FCS, PAA Laboratories) at 26°C. Cultures were set up initially at a density of 2.5×10^5^ parasites/ml and sub-passaged every 6 days.

### *Leishmania* extracts for metabolite analysis

*L. donovani* promastigote cultures were initiated at 2.5×10^5^ cells/ml in 16×10 ml cultures in order to obtain cell samples from four independently growing cultures (biological replicates) on each day. Promastigotes from each culture were harvested at days 3, 4, 5 and 6 for metabolite extraction. The metabolite extraction was performed as previously described [11]. Briefly, promastigotes quenching was performed in a dry ice/ethanol bath with rapid temperature decrease to 2°C and then immediate transfer to ice. Two aliquots of 4×10^7^ cells were taken from each culture flask (technical replicates). Cell pellets were obtained by centrifugation at 12000 g for 10 min at 4°C, and washed 3 times in 1 ml of PBS. For cell disruption and metabolite extraction, cell pellets were resuspended in 200 µl cold chloroform/methanol/water (20/60/20, v/v/v) and incubated for 1 h in a Thermomixer (1400 rpm, 4°C). After centrifugation at 12000 g for 10 min at 4°C, the supernatant containing the extracted metabolites was recovered and stored at −70°C until analysed.

### Liquid chromatography mass spectrometry (LC-MS) analysis and data processing

LC-MS analysis and data processing was done as described by t'Kindt and co-workers [11], [12]. Metabolite level comparisons between the time points analyzed (after 3, 4, 5 and 6 days of in vitro growth) were performed based on the ratio between the intensity on each day and the mean intensity level for the 4 day period, that is x/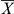
_3–6_. The following criteria were applied to assign differences in metabolite levels among the time points analyzed as being potentially interesting and so worthy of inclusion in the full analysis: (i) there was at least a 2-fold difference between at least one of the time points when compared with the mean intensity level; and (ii) there was a statistically significant difference (p<0.05) between the time points being compared. The data are expressed as intensity per 25 µg cell protein.

### Statistical analysis

Statistical analysis was performed using Analysis of Variance (ANOVA), which allowed the simultaneous comparison of all time point analyzed: a *p* value smaller than 0.05 (p<0.05) was considered significant; SPSS Statistics software (IBM) was used to perform principal component analysis; GraphPad Prism 4 was used for plotting the graphs and VisuMap software (VisuMap Technologies Inc.) was used to visualise the data as heatmaps.

## Results

**Figure 1:**
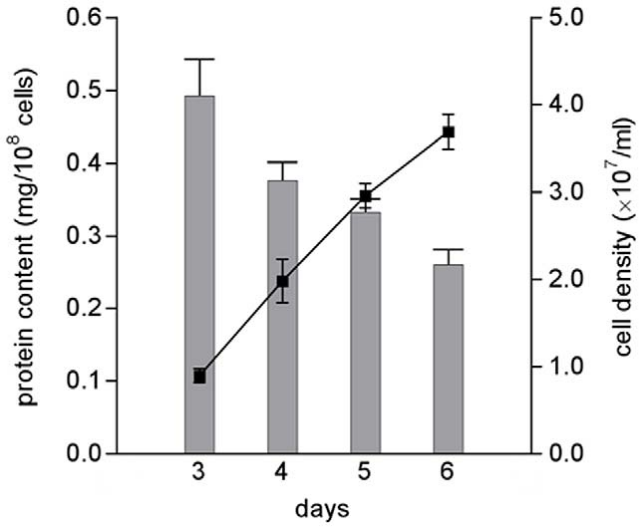
Relantionship between protein content and cell density during *in vitro* growth of *L. donovani* promastigotes. *L. donovani* promastigotes were seeded at 2.5×10^5^ parasites/ml, counted daily and harvested at days 3, 4, 5 and 6. Protein content was determined by the Lowry method. Each value is a mean ± standard deviation from four cultures. Statistically significant differences observed between the days evaluated: days 3 and 4, *p*<0.05; days 5 and 6, *p*<0.05; days 4 and 6, *p*<0.05; days 3 and 6, *p*<0.01.

In order to obtain detailed information about the metabolic changes that occur during the development of promastigotes under defined conditions in vitro, we have applied an untargeted metabolomics approach. Promastigotes were collected after 3, 4, 5 and 6 days of in vitro growth (thus including different proportions of various promastigote forms, including procyclic promastigotes which dominate during logarithmic growth and the non-dividing metacyclic forms that start to be formed at late stages of logarithmic phase) and analyzed by LC-MS. We used four parallel cultures (designated biological replicates) to obtain representative data and during the cell processing from each culture two samples were taken (designated technical replicates) to control for variation due to technical factors. Analysis of a parasite's metabolome needs to take into account the changes in cell volume that occur during development and growth. Measurement of the protein content of the cells showed that transformation to the metacyclic form at late logarithmic phase of growth was accompanied by a decrease in protein content, which is thought to correlate with the decrease in cell size (Figure 1). There was a great difference in protein content between the cells on days 3 and 6 (p<0.0028) but also a significant difference between the cells on days 3 and 4 (p<0.025) and days 5 and 6 (p<0.034). To assure that the metabolome analysis of the parasite would reflect the changes observed in cell size, we have expressed the data as intensity per cell protein (rather than per cell number), thus facilitating meaningful comparison of the metabolite levels in cells of differing volume. This method of normalization had significant effects, for instance the general decreases in metabolite intensity when expressed per cell number that were observed between promastigotes on day 3 and day 6 were almost abolished when data were expressed as intensity/mg cell protein. Indeed the summed total of the metabolite intensities normalized to cell protein were relatively constant over the four days whereas when expressed as intensity/cell number it declined 33% on day 6. We believe that this method of taking into account the changes in cell size during growth is currently optimal and provides a means of generating data that are meaningful and can be interpreted with confidence.

**Figure 2:**
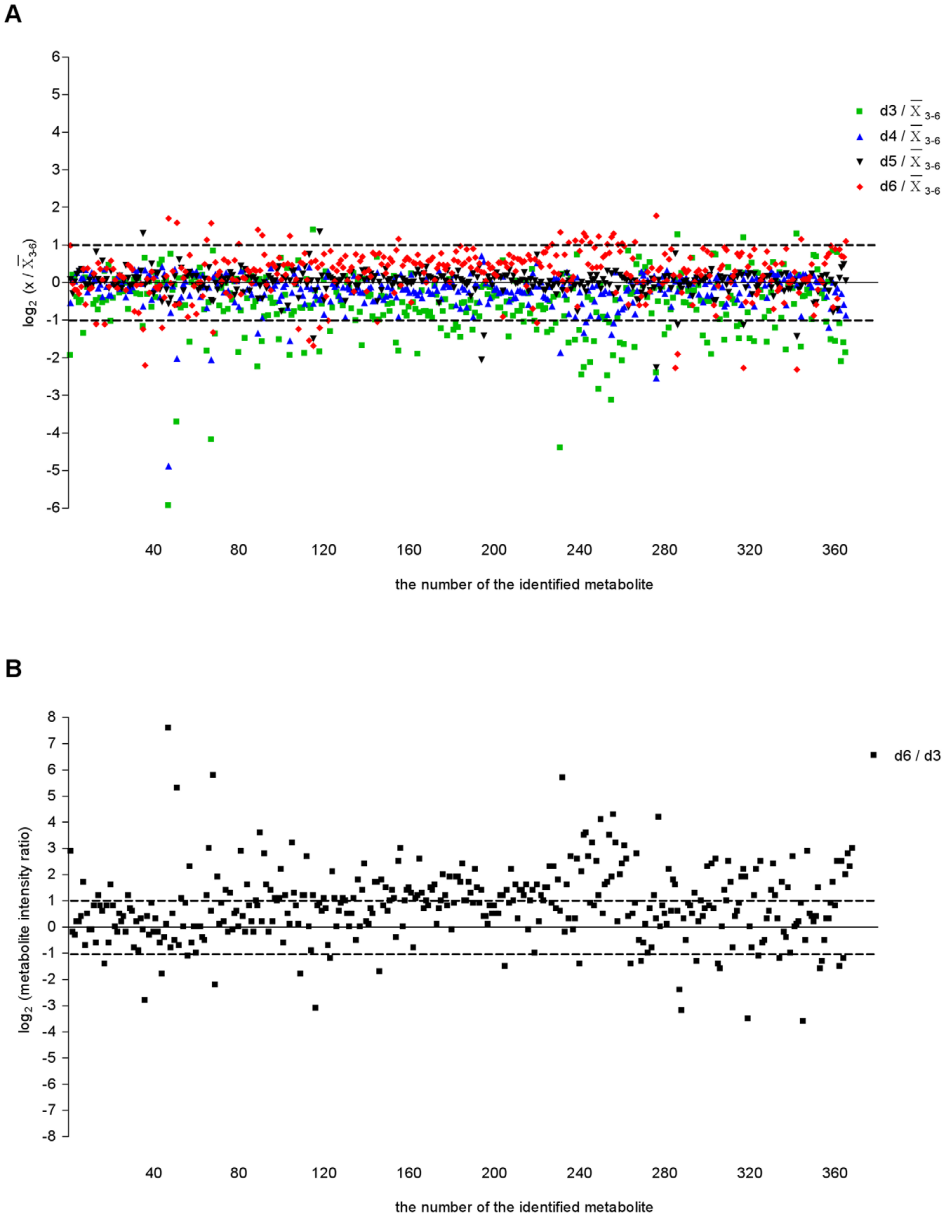
Overview of intracellular metabolite levels in *L. donovani* promastigotes during *in vitro* growth. (A) Relative levels of intracellular metabolites in *L. donovani* promastigotes detected from day 3 to day 6. Metabolite intensity levels on each day were compared with the mean level over the 4-day period. Following a logarithmic transformation (base 2), the relative level of each metabolite was plotted. Dotted lines represent the fold-change cut-off considered as significantly different between the day analyzed and the mean level of the 4-day period: log_2_ (dx/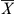
_3–6_) below or above 1 indicates a 2-fold change; dx, time point analyzed (day 3, 4, 5 or 6). The metabolites differing at least by 2-fold on one of the days when compared with the mean were 26.9% of the all of the metabolites identified. (B) Comparison between day 6 (d6) and day 3 (d3) intensity ratios for each identified metabolite. All metabolites within the 2 ppm cut-off are included and following a logarithmic transformation (base 2) the average signal intensity ratios of d6/d3 for all metabolites were plotted. Dotted lines represent the fold-change cut-off in this study for a given metabolite profile being considered to be significantly different between day 6 and day 3: log_2_ (metabolite intensity ratio) below −1 or above 1 indicates a 2-fold change between time points compared. The metabolites differing at least by 2-fold between day 6 and day 3 were 48.4% of the total number of metabolites identified.

**Figure 3:**
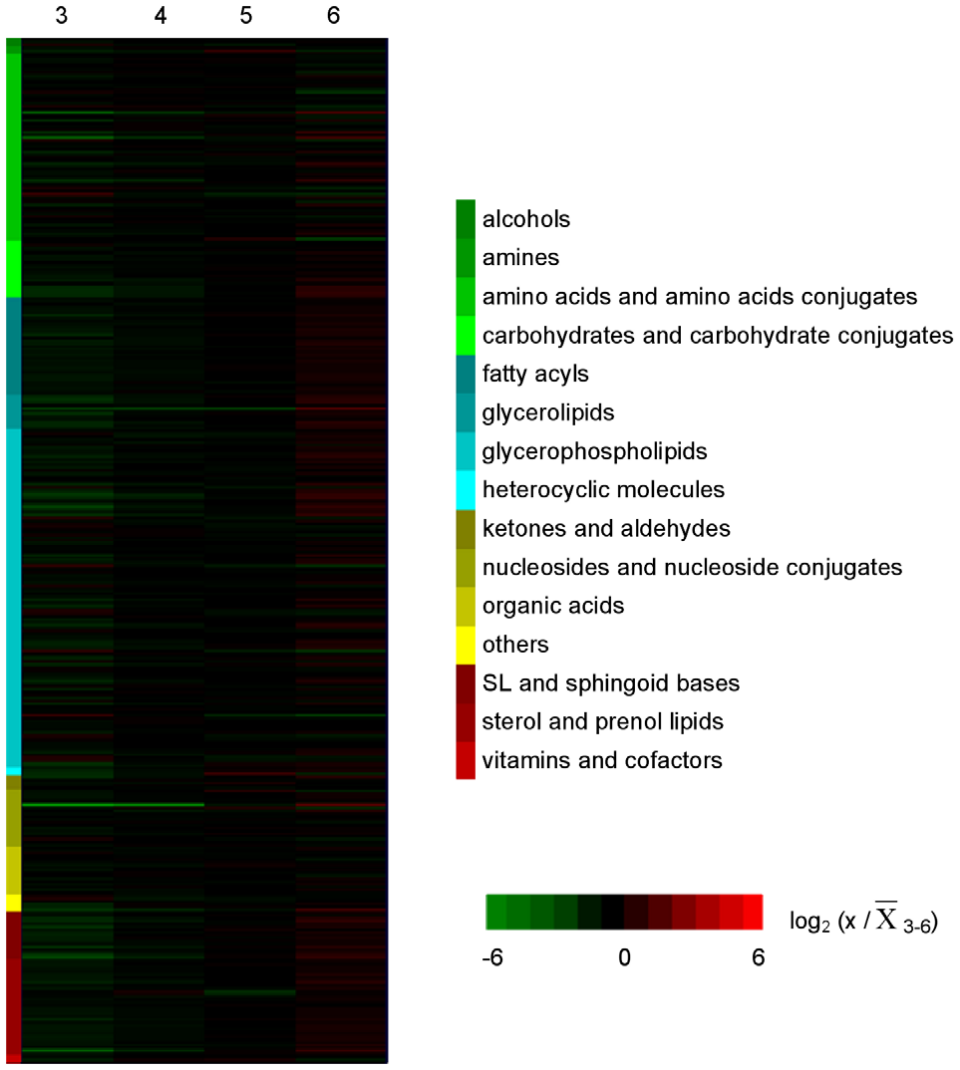
Intracellular metabolic fluctuations during *in vitro* development of *L. donovani* promastigotes. Profiles of all intracellular metabolites identified below 2 ppm deviation in relative levels. Metabolite levels on each day were compared with the mean level over the 4-day period. Following a logarithmic transformation (base 2), the relative level of each metabolite is shown; log_2_ (x/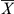
_3–6_) below −1 or above 1 indicates a 2-fold change; x, time point analyzed (day 3, 4, 5 or 6). Metabolite profiles are ordered by compound category shown on the left and log values of measurements are color-coded as indicated in the scale on the right of the heat map, from green to red.

In order to understand better the metabolic fluctuations as the promastigotes developed over the four days, we compared the profiles of metabolites levels (Figures 2A and 3). The analysis shown in Figure 2A (in which the metabolite intensity level on each day is compared with the mean level over the 4 day period) highlights that, of the total metabolites identified, the levels of the majority remained rather similar throughout although 26.9% differed by at least 2-fold on one of the days when compared with the mean. The day 3 levels were the most different from the others (22% being at least 2-fold different from the centered-mean for the period analyzed) and with just some metabolites differing greatly at other times. This is consistent with there being a progressive change in many metabolite levels over the four day period. However, comparison between the metabolite intensities on days 3 and 6 revealed that 48.4% of all the metabolites identified differed by more than 2-fold (Figure 2B), suggesting a significant difference in metabolic profile between promastigote populations in logarithmic (mainly procyclic promastigotes) and stationary phases (containing many metacyclic promastigotes).

**Figure 4:**
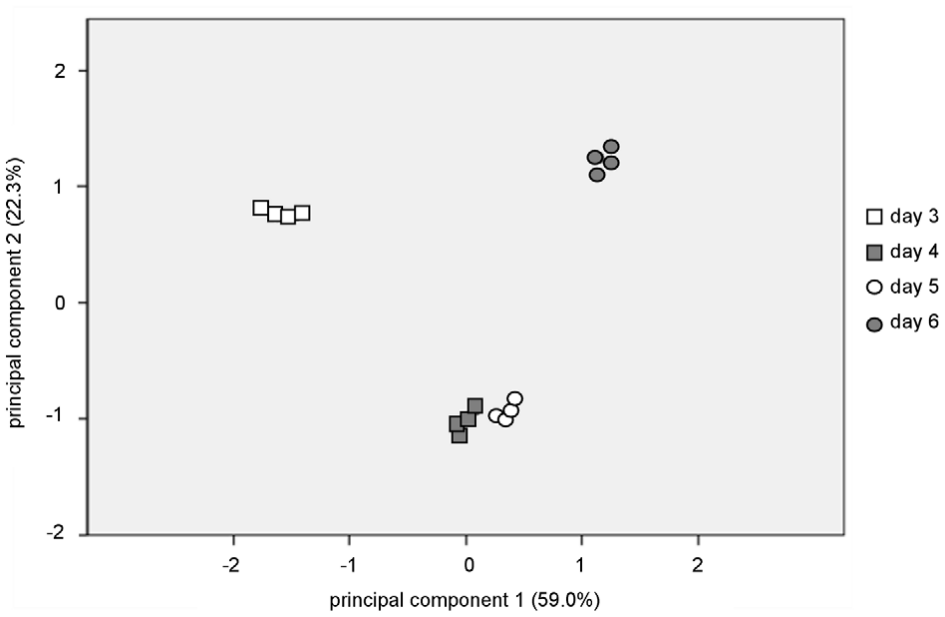
*L. donovani* promastigote development is accompanied by significant changes in the metabolome. Principal component analysis of all metabolites identified by LC-MS in cell extracts from *L. donovani* promastigotes harvested on days 3, 4, 5 and 6 of in vitro growth; each datapoint corresponds to the analysis of one biological replicate. Principal component 1 and principal component 2 explain 59.0% and 22.3% of the total variance, respectively.

The metabolic profile was also analysed by principal component analysis (PCA). PCA is an unsupervised clustering technique that allows the reduction of the data into two dimensions (principal component 1 [PC1] and principal component 2 [PC2]), which capture and enable visualization of data variability; this method is generally applied to large sets of data, such as those resulting from microarray or metabolomic analyses, as a way of obtaining a summary or overview of all samples, to find clusters and trends, and to identify the outliers. It is recommended as a starting point for analysis of multivariate data [22]. The PCA score plots (Figure 4) of the LC-MS data show clearly the identification of four distinct clusters, each one corresponding to one of the groups of samples analyzed on a particular day of growth. PC1 and PC2 account for more than 81% of the variables which shows the clear metabolic differences between the samples. Moreover, the tight clustering within each group indicates good reproducibility. The data in Figure 4 show that promastigotes on days 4 and 5 are aligned closely with each other indicating that they have a similar metabolic profile that is clearly distinguished from those on days 3 and 6 (which explains 59.0% of total variance given by the second principal component). These data are consistent with there being metabolic changes as the promastigotes develop from procyclic promastigotes to metacyclic promastigotes, and the relatively large number of metabolites that differ in levels significantly between days 3 and 6.

The identity of the metabolites was carried out based on the databases detailed by t'Kindt and co-workers [11], [12]. We were able to identify 368 putative metabolites (267 at <1 parts per million [ppm] deviation and 101 at the 1–2 ppm deviation level). The compounds identified belong to a wide range of metabolic pathways and include amino acids, nucleosides, carbohydrates, fatty acyls, sterols and glycerophospholipids among others, as shown in Figure 3. The full list of putatively identified metabolites at days 3, 4, 5 and 6 at below 1 ppm and between 1–2 ppm deviation are provided in Tables S1 and S2 of supplementary data, respectively. The majority of the metabolites remained at a relatively constant level. Indeed, the overall sum of intensities of the identified metabolites in the samples from the different days show that there is little apparent variation in the total metabolome identified, with the only difference being between day 3 and day 4 (Figure S1); clearly, however, such data have to be used with caution as not all of the parasite's metabolites are included in the dataset and the method is not fully quantitative. There were, however, some apparent variations within each group of metabolites (Figure S2). Lipids, in general, increased substantially from day 3 to day 6. Carbohydrates and nucleosides similarly apparently increase in abundance, whereas other groups of metabolites including amino acids and derivatives, organic acids and alcohols remain at relatively constant levels.

**Figure 5:**
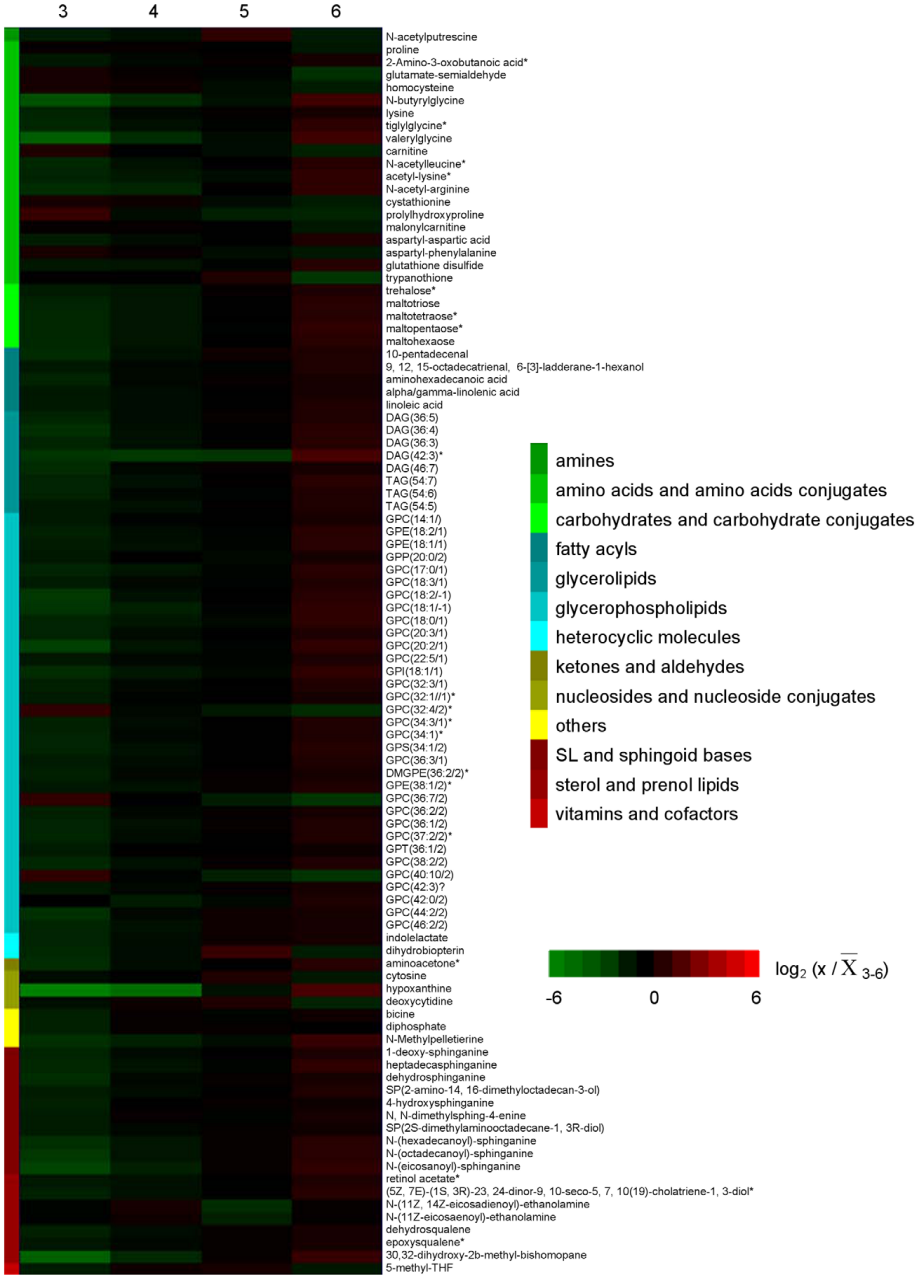
Heatmap depicting significant fluctuations in *L. donovani* promastigotes intracellular metabolome during *in vitro* development. Profiles over 4 days of intracellular metabolites that differed at least by 2-fold from the mean levels in at least one of the time points analyzed. Metabolite intensity levels on each day were compared with the mean level over the 4 day period. Following a logarithmic transformation (base 2), the relative level of each metabolite is shown; log_2_ (x/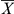
_3–6_) below −1 or above 1 indicates a 2-fold change; x, time point analyzed (day 3, 4, 5 or 6). Metabolites labelled with * represent peaks with multiple potential identifications, for which just one is shown in this figure but the full list is given in the Supporting Information; metabolite profiles are ordered by compound category shown on the left and log values of ratios are color-coded as indicated in the scale on the right of the heat map, from green to red.

All metabolites that differed from the mean for the 4-day period by at least a 2-fold on one or more days and were statistically different between the time points analyzed (*p*<0.05) are represented in heatmap format to visualize the main changes that occur during transformation of promastigotes in logarithmic phase to those in stationary phase (Figure 5) and the intensity levels are provided on Table S3. This group (97 in total, 26% of the total number of metabolites putatively identified) includes metabolites representative of all of the compound categories shown in Figure 3 with the exception of organic acids and alcohols. It was possible to distinguish five general patterns by which metabolites fluctuated during the 4-day period analyzed (Figure S3 and Table S3). The levels of some metabolites continually increased from day 3 to day 6 (pattern 1, 74% of the 97 varying metabolites), while the opposite happened with others (pattern 3, 9%). Other metabolites showed peak levels on days 4 or 5 which then declined (pattern 2, 9%), while others decreased from day 3 to day 4 and then increased (pattern 4, 2%). Some metabolites had a fluctuating profile showing an increase followed by a decrease and then another increase (pattern 5, 6%).

**Figure 6:**
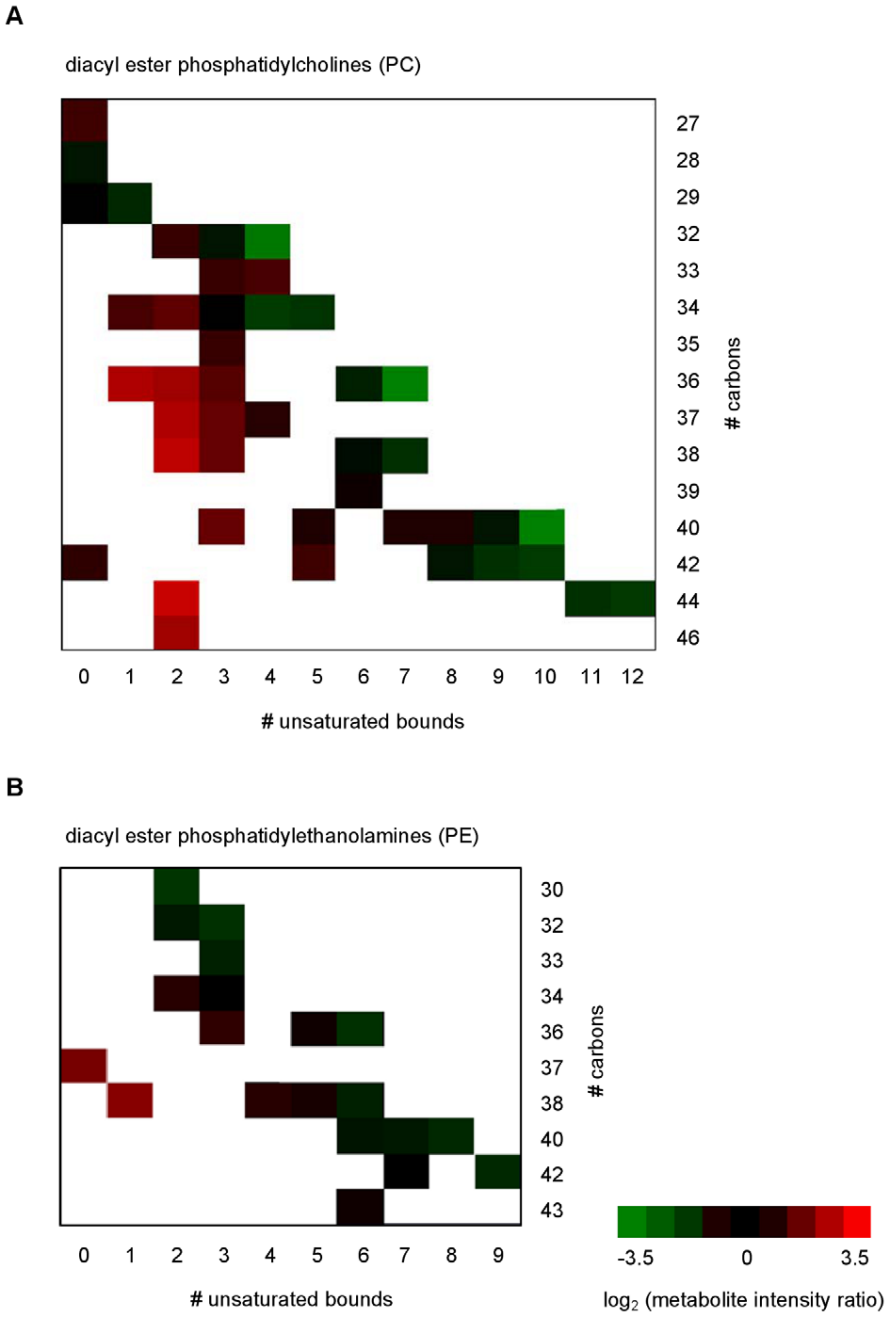
Comparative analysis of phosphatidylcholines and phosphatidylethanolamines in *L. donovani* promastigotes on days 3 and 6. Fatty acyl structure properties in (A) phosphatidylcholines (PC) and (B) phosphatidylethanolamines (PE) for each phospholipid detected on day 6 and day 3. The x-axis shows the total number of unsaturated bonds present in the 2 fatty acyl chains, while the y-axis shows the length of the fatty acyl chains in total number of carbon units. Data are shown as ratios of signal intensity, day 6/day 3, after a logarithmic transformation (base 2) and represented by a color code as indicated in the scale on the right of the heat map, from green to red, where green represents a decrease and a red an increase in abundance of the given phospholipid on day 6.

**Figure 7:**
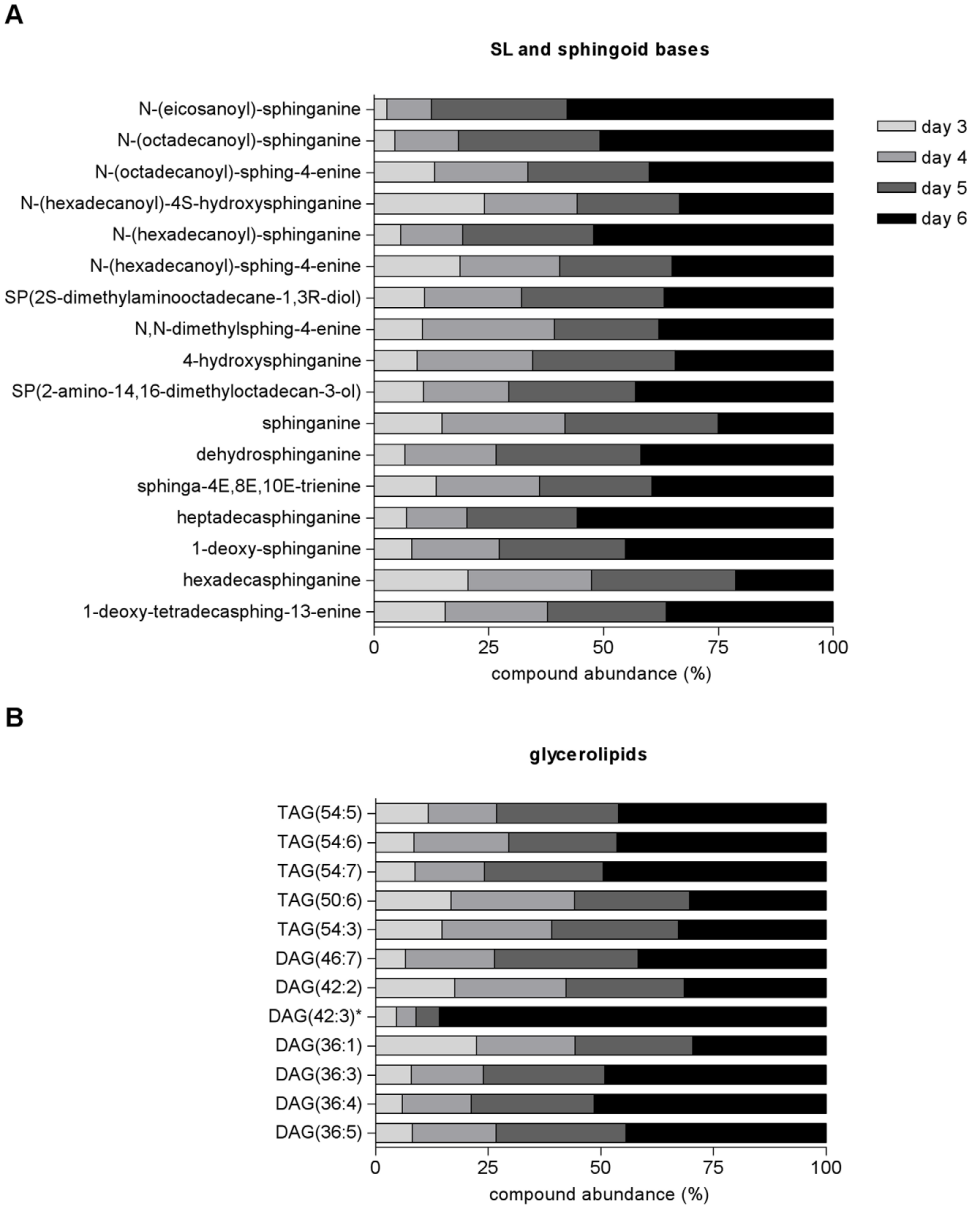
Stage specific increase of sphingolipids and glycerolipids abundance during *L. donovani* promastigotes development *in vitro*. (A) Sphingolipids and sphingoid base and (B) glycerolipid metabolites abundance is represented for each day as a percentage of the total abundance detected, which comprises the sum of the total metabolite intensity on each of the 4 days analysed. Metabolites labelled with * represent peaks with multiple potential identifications, for which just one is shown in this figure but the full list is given in the Supporting Information. DAG, diacylglycerol; TAG, triacylglycerol.

A more detailed analysis of each of the categories of metabolites suggests specific variation potentially related with the cell stage. For instance, analysis of structural properties of the fatty acyl side chains of phosphatidylethanolamine (PE) and phosphatidylcholine (PC) lipids revealed that there was an increased abundance of the PC lipids with lower unsaturated fatty acyl chains as the promastigotes developed from day 3 to day 6 (Figure 6 and S4 in supplementary data); this seems also to happen in PE lipids, but less so than with PC lipids. These data suggest that there are changes in the composition of membranes with development from procyclic to metacyclic promastigotes. Another class of metabolites showing striking differences depending on the cell stage were the sphingolipids (SLs). In *Leishmania*, SLs are not essential for growth but they are for differentiation, probably due to the high demand in vesicular trafficking required for parasite remodeling [23]. The abundance of these metabolites in general increased on day 5 and greatly on day 6, such that for some of the SLs identified, such as N-(eicosanoyl)-sphinganine, N-(hexadecanoyl)-sphinganine and heptadecasphinganine, the day 6 intensity amounted to more than 70% of the total amount detected over the four days (Figure 7A). A similar situation was seen with some of the identified glycerolipids, with the diacylglycerol putatively identified as DAG(42∶3) being especially increased on day 6 (Figure 7B). The abundance of sterols, prenol lipids and fatty acyl metabolites generally increased during growth and thus were more abundant on days 5 and 6, although there were exceptions such as N-(11Z-eicosaenoyl)-ethanolamine and N-(11Z,14Z-eicosaenoyl)-ethanolamine (Figures S5 and S6).

In contrast to the lipids, amino acids and derivatives in general did not differ greatly during the four day period, although some were less abundant on day 6 (proline, glutamate-semialdehyde, homocysteine, carnitine and cystathionine among others) while others were increased (for example N-butyrylglycine, lysine, valerylglycine, acetyl-lysine, and N-acetyl-arginine) (Table S3 and Figure S7). A higher abundance of carbohydrates, such as maltohexose among others, was observed as cells reach stationary phase (Table S3 and Figure S8). The intensity variation for hypoxanthine and xanthine over the 4 days had a clearly distinct pattern from the other nucleosides or nucleoside conjugates, with a large increase in the abundance of these metabolites on day 6, while, for example, cytosine and deoxycytidine decreased in abundance on day 6 (Table S3 and Figure S9). The great increase in the abundance of hypoxanthine and xanthine at day 6 is responsible for the large change observed in the overall abundance of all metabolites included in this group (Figure S2); the overall abundance of this group was relatively unchanged over the 4 day period of analysis if these two metabolites were not included. None of the metabolites included in the organic acids group accomplish the criteria defined, despite, for example, the statistically significant difference observed in the levels of mevalonate on day 6 (Figure S10). A marked decrease in abundance on day 6 was also observed for 5-methyl-THF, dihydrobiopterin and N-acetylputrescine (Table S3 and Figure S11).

## Discussion

*Leishmania* promastigotes development in the sand fly includes a wide range of modifications in order to prepare the parasite for transmission to a mammalian host. The number of distinct developmental stages that occur is uncertain for although many have been named and identified based on morphology [3], [4] most have not been sufficiently characterized to be certain that they are truly distinct developmental stages. As expected, in vitro we were able to observe various morphological forms of *L. donovani* promastigotes but the major clear difference was the appearance of small morphs as the culture reached stationary phase, when metacyclic forms are predominant. This reflects the remodeling of cell shape during life cycle transitions and involved a decrease in protein content (Figure 1). This was taken into consideration in analyzing the metabolite dataset and indeed the data were normalized to protein content as a means of taking into account changes in cell size.

Analysis of metabolome during in vitro growth of promastigotes revealed that whereas the overall metabolite abundance remained relatively constant there were variations in the levels of individual metabolites, suggesting that parasite differentiation from procyclic to metacyclic forms takes place in a progressive manner and involves changes in certain individual or groups of metabolites. This study reinforces the idea that there are multiple forms of promastigotes that are adapted differently at the metabolic level, presumably reflecting the differing challenges that they face naturally in the sand fly. It has been postulated previously from studies on morphology of *Leishmania* promastigotes in sand flies and in vitro cultures that the parasite undergoes similar developmental transitions in vitro as occur in the sand fly host [24], despite the absence of the host pressure. This has been interpreted as the parasite being genetically pre-adapted to survive in the sand fly. The biochemical changes that accompany these morphological/developmental changes are not fully known, although some characteristics of the metacyclic promastigote of *L. major* have been reported. Differentiation to the infective metacyclic promastigote form involves modifications in LPG structure [6], [25], which have been shown to occur both in in vitro culture and during in vivo development in the sand fly [26]. HASPB and SHERP are stage-specific proteins present in the infective stages, with SHERP being exclusively present in the metacyclic forms; the stage-specific expression of both has been observed in vitro as well as during the development in the vector [8]. Other surface molecules, including the metallopeptidase GP63, also undergo changes in expression pattern as the promastigotes development progresses [27]. The LPG modifications are essential for parasite infectivity and occur in vitro and in vivo demonstrating that in the absence of the host this essential processes still occurs; these findings are suggestive that other changes similarly also occur in vitro and the data of our current study show that indeed this is the case.

Metacyclogenesis is marked by a great increase in membrane trafficking and remodeling [28] and previous studies have shown that the organization of *Leishmania* membrane differs between procyclic and metacyclic promastigotes, in part due to the distribution of LPG into lipid rafts during differentiation [29]. Phospholipids (PLs) account for ∼70% of total cellular lipids in *Leishmania*, with PC, the most abundant glycerophospholipid, predominantly present in a diacyl form [30] with unusually long and unsaturated fatty acid species [31]. These properties, the acyl length and degree of unsaturation, may play an important role in the fluidity of *Leishmania* membrane, thus they are likely to be regulated throughout parasite development in its hosts. Indeed, our data show that promastigote phospholipid composition changed remarkably in terms of the unsaturation levels observed in the fatty acid chains, in particular of PC lipids. Promastigotes in stationary phase (day 6) presented a higher abundance of PC lipids with lower levels of unsaturation than those observed on day 3 (Figure 6), revealing a shift towards lower unsaturation of PC lipids and consequently a decrease in membrane fluidity with metacyclic promastigote generation. These observed changes in membrane fluidity may be a mechanism whereby the parasite becomes pre-adapted for survival upon infecting a mammalian host, at which time it is confronted by a dramatic increase in temperature. Thus perhaps the change in membrane composition enables the parasite to maintain an appropriate membrane fluidity even at the higher temperature encountered. It is well known that the well-being of organisms is dependent upon the maintenance of optimal level of membrane fluidity [32]. In yeast, changes in the degree of unsaturation of fatty acids has been reported as a response to changes in the environmental temperature and complements other mechanisms such as modifications in fatty acid chain length, branching and cellular fatty acid content [33]. A recent study by Turk and co-workers [34] have related membrane fluidity to the adaptation level of different yeast to environmental stresses and to their growth temperature range, demonstrating that plasma-membrane fluidity can be used as an indicator of fitness for survival in extreme environments [34]. Changes in membrane fluidity in plants was also suggested to be crucial in sensing and influencing gene expression during temperatures fluctuations [35]. Alterations in membrane fluidity have been associated with the occurrence of drug resistance in *Leishmania*; it is thought that membrane lipid composition may influence drug-membrane interactions and interfere with drug uptake by the amastigotes residing in the mammalian host [36]–[38]. Indeed, comparison of promastigotes derived from clinical *L. donovani* isolates with different antimonial sensitivity has shown a shift towards higher unsaturation of PC lipids in drug-resistant clones, suggesting an increase in membrane fluidity that may be related to the changes in uptake ability observed in the drug-resistant cell lines [12].

Another group of lipids that notably increased in abundance during *L. donovani* promastigotes development in vitro were the SLs (Figure 7A). SLs are not required for growth of *Leishmania*, since parasites that completely lack SLs grew normally in logarithmic phase and were still able to make “lipid rafts”. However, deletion of *spt2-*, the gene that encodes the key de novo biosynthetic enzyme serine palmitoyltransferase subunit 2, resulted in parasites deficient in de novo SLs synthesis that once in the stationary phase were not able to differentiate into metacyclic forms [23]. The increase in SLs during stationary phase we have found in this study is consistent with the requirement of these metabolites for differentiation to metacyclic froms. SLs are considered essential membrane components in all eukaryotes, mediating many signaling pathways including those key for apoptosis, growth and differentiation [39]. However, in *Leishmania* the primary role of SLs appears to be the provision of ethanolamine, as ethanolamine supplementation was able to overcome the phenotype observed in the SL-deficient mutant parasites [40]. Ethanolamine and choline are essential nutrients, and when available exogenously they can be salvaged by *Leishmania* via membrane transporters [41], [42]. Thus the significance of SL biosynthesis is likely to be stage-specific, being important in those stages in the sand fly that cannot rely upon salvaged ethanolamine. Indeed, amastigotes deficient in de novo SLs synthesis recovered from a mammalian host showed normal levels of inositolphosphoryl ceramide (IPC) and thus amastigotes seems to be able to perform SLs salvage [43].

Glycerolipids, represented by diacylglycerols (DAG) and triacylglycerols (TAG), also increased in abundance as *L. donovani* promastigote development progressed (Figure 7B). PC and PE lipids are synthesized by conjugation of a lipid anchor such as DAG with either CDP-choline or CDP-ethanolamine, the last step of the in de novo biosynthesis of phospholipids (the Kennedy pathway) (reviewed in [44]). Thus the observed increase in neutral lipids correlates well with the changes observed in *Leishmania* membrane lipid composition during promastigote development.

Sterol and prenol lipids also increased with time in culture, although the changes during *L. donovani* promastigotes development were not so accentuated as for SLs and glycerolipids. Sterols are the target of the important antileishmanial drug amphotericin B [36] and they may also play a significant role in the activity of miltefosine against the parasite, as sterol depletion led to a decrease in susceptibility [45]. Effectiveness of these drugs is mainly dependent on their interaction with the *Leishmania* membrane, thus it is clear that the ability of the parasite to change its lipid membrane composition, which occurs inherent during its life cycle, should be taken into consideration when considering new drug formulations.

*Leishmania* parasites are auxotrophic for many amino acids and must scavenge essential amino acids from their hosts. Besides the use in protein biosynthesis, some amino acids, notably proline, can be used as major energy sources [46]. Recently, Saunders and co-workers have reported that aspartate, alanine and glutamate are internalized by *L. mexicana* promastigotes and incorporated into the TCA cycle, revealing the importance of this pathway in glutamate, glutamine and proline synthesis and demonstrating that the TCA cycle in *Leishmania* is not only a catabolic pathway [47]. One notable feature in the levels of amino acids and amino acid conjugates during growth of promastigotes in vitro was a large increase in metabolites of fatty acids named acyl glycines (valerylglycine, tiglyglycine, N-butyrylglycine). Increases in levels of acyl glycines in higher eukaryotes is associated with mitochondrial energy metabolism disorders, indeed the measurement of these metabolites is used as a diagnostic tool. Glycine conjugation is considered to be an important detoxification system, preventing the accumulation of acyl-CoA esters in several inherited metabolic disorders of humans [48]. Moreover, valerylglycine was found to be increased in urine of *Plasmodium vivax*-infected individuals, which indicates an alteration in the fatty acid metabolism during infection [49]. These changes in acylglycines abundance during promastigote development could reflect changes in mitochondrial metabolism, but caution needs to be exercised as increased levels of this group of compounds was also found in drug-resistant parasites [12] and also in genetically manipulated mutants (A.M. Silva et al., unpublished results) – which indicates that the levels of these metabolites may be disturbed in a variety of situations.

*Leishmania* also take up from their environment other essential nutrients, such as purines and growth factors. Biopterin and folate uptake has been shown to decrease when promastigote have entered stationary phase [50], which is consistent with our findings that there was a decrease in abundance of 5-methyl-THF and dihydrobiopterin in *L. donovani* promastigotes at day 6 of in vitro growth. Indeed, low levels of tetrahydrobiopterin were associated with increased differentiation of *L. major* into the infective metacyclic form and thus postulated to be an important factor controlling this process [51]. *Leishmania* parasites are not able to synthesize purines de novo and need to acquire either nucleosides or nucleobases [52]. Hypoxanthine uptake by *L. major* promastigotes is greatly reduced in stationary phase compared with logarithmic growth phase. This was shown to correlate with down-regulation of expression of the NT3 permease as promastigotes reach stationary phase [53] and it was reasoned that these changes reflected the fact that at this stage the population is mainly composed by non-dividing cells, the metacyclic promastigotes, that do not require purines for mitosis. Our findings of an increase in the levels of hypoxanthine and xanthine at day 6 of *L. donovani* promastigotes in vitro growth explain why less uptake is required at this stage. Moreover, one can speculate that these higher levels in the metacyclic forms are beneficial in enabling the subsequent differentiation events after infection of a mammal, a transition phase that occurs in the parasitophorous vacuole of a macrophage where availability of some nutrients may be limiting [54], [55].

Overall the results of this study have provided convincing data that promastigotes of *Leishmania* at different stages of culture in vitro differ from each other significantly in terms of the composition of their metabolome, whereas the total metabolite abundance appears to remain relatively constant as the promastigotes develop from day 3 to day 6. The study has provided insights into the overall changes that occur, which adds to the many previous reports on changes in individual metabolites, groups of metabolites and enzymatic reactions involved in metabolite production (see, for example, [16], [46], [56]–[58]). Our data are consistent in particular with previous findings obtained using other approaches, such as changes observed in the content of sphingolipids and other lipids that may contribute to successful parasite survival in the mammalian host [23], [57]. These changes observed undoubtedly reflect adaptations to differing conditions that *Leishmania* encounters in its two hosts, but the full understanding of how these adaptations function require additional data on the environments themselves (the detailed content of the parasitophorous vacuole in a macrophage and the intestinal tract of the sand fly, and how these change with time, are largely unknown) as well as more complete analyses of metabolism of individual promastigote forms and if possible integration of the generated data with those arising from other –omics approaches. However, understanding the variation in metabolism of promastigotes will be informative in elucidating more fully the metabolic capabilities of *Leishmania* and hopefully highlight unusual features that can be exploited in novel approaches to designing therapies.

## Supporting Information

Figure S1 **Overall metabolite levels remain similar during** ***L. donovani*** **promastigotes development** ***in vitro*** **.** Sum of total metabolite intensities from *L. donovani* promastigotes identified by LC-MS analysis at days 3, 4, 5 or 6 of in vitro growth.
(PDF)

Figure S2 **Overall metabolite levels for each compound category during** ***L. donovani*** **promastigotes development** ***in vitro*** **.** Sum of total metabolite intensities from *L. donovani* promastigotes identified by LC-MS analysis at days 3, 4, 5 or 6 of in vitro growth grouped by compound categories.
(PDF)

Figure S3
**Patterns of metabolite variation in level during** ***L. donovani*** **promastigote** ***in vitro*** **growth from day 3 to day 6.** Analysis of *L. donovani* promastigotes metabolome at day 3, 4, 5 and 6 allowed the distinction of 5 patterns (1–5) based on the metabolic profile for each metabolite that differed in intensity by 2-fold or more from the mean levels in at least one of the time points analyzed (a total of 97 metabolites). The relative level of each metabolite is shown, following logarithmic transformation (base 2); log_2_ (x/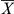
_3–6_) below −1 or above 1 indicates a 2-fold change; x, time point analyzed (day 3, 4, 5 or 6). (A) Pattern 1 (74% of all metabolites considered) and (C) pattern 3 (9%) include metabolites that increased or decreased, respectively, continuously during all the whole period analyzed; (B) pattern 2 (9%) include metabolites which had peak levels on days 4 or 5 but which then declined; (D) pattern 4 (2%) metabolite levels decreased from day 3 to day 4 and then increased and (E) pattern 5 (6%) include metabolites that showed an increase followed by a decrease and then another increase.(PDF)

Figure S4 **Glycerophospholipids abundance during** ***L. donovani*** **promastigotes development** ***in vitro*** **.** Glycerophospholipids abundance is represented for each metabolite on each day as a percentage of the total abundance detected, which comprises the sum of the total metabolite intensity during the 4-day period of analysis.
(PDF)

Figure S5 **Sterol and prenol lipids abundance during** ***L. donovani*** **promastigotes development** ***in vitro*** **.** Sterol and prenol lipids abundance is represented for each metabolite on each day as a percentage of the total abundance detected, which comprises the sum of the total metabolite intensity during the 4-day period of analysis. Metabolites labelled with * represent peaks with multiple potential identifications, for which just one is shown in this figure but the full list is given in Tables S1 and S2.
(PDF)

Figure S6 **Fatty acyls abundance during** ***L. donovani*** **promastigotes development** ***in vitro*** **.** Fatty acyls abundance is represented for each metabolite on each day as a percentage of the total abundance detected, which comprises the sum of the total metabolite intensity during the 4-day period of analysis. Metabolites labelled with * represent peaks with multiple potential identifications, for which just one is shown in this figure but the full list is given in Tables S1 and S2.
(PDF)

Figure S7 **Amino acids and amino acids conjugates abundance during** ***L. donovani*** **promastigotes development** ***in vitro*** **.** Amino acids and amino acids conjugates abundance is represented for each metabolite on each day as a percentage of the total abundance detected, which comprises the sum of the total metabolite intensity during the 4-day period of analysis. Metabolites labelled with * represent peaks with multiple potential identifications, for which just one is shown in this figure but the full list is given in Tables S1 and S2.
(PDF)

Figure S8 **Carbohydrates and carbohydrate conjugates abundance during** ***L. donovani*** **promastigotes development** ***in vitro*** **.** Carbohydrates and carbohydrate conjugates abundance is represented for each metabolite on each day as a percentage of the total abundance detected, which comprises the sum of the total metabolite intensity during the 4-day period of analysis. Metabolites labelled with * represent peaks with multiple potential identifications, for which just one is shown in this figure but the full list is given in Tables S1 and S2.
(PDF)

Figure S9 **Nucleosides and nucleoside conjugates abundance during** ***L. donovani*** **promastigotes development** ***in vitro*** **.** Nucleosides and nucleoside conjugate metabolites abundance is represented for each metabolite on each day as a percentage of the total abundance detected, which comprises the sum of the total metabolite intensity during the 4-day period of analysis. Metabolites labelled with * represent peaks with multiple potential identifications, for which just one is shown in this figure but the full list is given in Tables S1 and S2.
(PDF)

Figure S10 **Organic acids abundance during** ***L. donovani*** **promastigotes development** ***in vitro*** **.** Organic acids abundance is represented for each metabolite on each day as a percentage of the total abundance detected, which comprises the sum of the total metabolite intensity during the 4-day period of analysis. Metabolites labelled with * represent peaks with multiple potential identifications, for which just one is shown in this figure but the full list is given in Tables S1 and S2.
(PDF)

Figure S11 **Vitamins and cofactors, ketones and aldehydes, heterocyclic molecules, amines and alcohols abundance during** ***L. donovani*** **promastigotes development** ***in vitro*** **.** Vitamins and cofactors, ketones and aldehydes, heterocyclic molecules, amines and alcohols abundance is represented for each metabolite on each day as a percentage of the total abundance detected, which comprises the sum of the total metabolite intensity during the 4-day period of analysis. Metabolites labelled with * represent peaks with multiple potential identifications, for which just one is shown in this figure but the full list is given in Tables S1 and S2.
(PDF)

Table S1 **Metabolites identified below 1 ppm deviation in samples of** ***L. donovani*** **promastigotes from day 3 to day 6 of** ***in vitro*** **growth.** For each compound the following information is shown: ionisation (ESI) mode; detected mass; retention time (min); putative metabolite identification; ppm deviation between detected mass and theoretical mass of the putative metabolite identified; intensity per 25 µg cell protein for each sample; average intensity per 25 µg cell protein in each time-point analyzed (3D, 4D, 5D or 6D); standard deviation in each time point analyzed; log base 2 of ratio of the average signal intensity in each time point against the mean intensity level during the 4 day period; F test statistics and p values obtained by analysis of variance (ANOVA), indicating whether or not there was a statistically significant difference between the time points analyzed; decision on whether or not the compound was significantly changed in level based on a two-fold or higher average difference in signal intensity in at least one of the time points analyzed and statistical significance (*p*<0.05); compound category.
(XLS)

Table S2 **Metabolites identified between 1–2 ppm deviation in samples of** ***L. donovani*** **promastigotes from day 3 to day 6 of** ***in vitro*** **growth.** The data presented are as described for Table S1.
(XLS)

Table S3 **Intensity levels of metabolites that differ significantly during** ***L. donovani*** **promastigote development** ***in vitro*** **.** Metabolites are ordered by compound category; for each metabolite is shown the average intensity per 25 µg cell protein at days 3, 4, 5 and 6 and the respective metabolic profile pattern (analysis shown in Figure S2). Metabolites labelled with * represent peaks with multiple potential identifications, for which just one is shown in this figure but the full list is given in Table S1 and S2.
(PDF)

## References

[1] Kedzierski L (2010). Leishmaniasis Vaccine: Where are We Today?. J Glob Infect Dis.

[2] Croft SL, Sundar S, Fairlamb AH (2006). Drug resistance in leishmaniasis.. Clin Microbiol Rev.

[3] Gossage SM, Rogers ME, Bates PA (2003). Two separate growth phases during the development of Leishmania in sand flies: implications for understanding the life cycle.. Int J Parasitol.

[4] Kamhawi S (2006). Phlebotomine sand flies and Leishmania parasites: friends or foes?. Trends Parasitol.

[5] McConville MJ, Blackwell JM (1991). Developmental changes in the glycosylated phosphatidylinositols of Leishmania donovani. Characterization of the promastigote and amastigote glycolipids.. J Biol Chem.

[6] McConville MJ, Turco SJ, Ferguson MA, Sacks DL (1992). Developmental modification of lipophosphoglycan during the differentiation of Leishmania major promastigotes to an infectious stage.. EMBO J.

[7] Sacks DL, Pimenta PF, McConville MJ, Schneider P, Turco SJ (1995). Stage-specific binding of Leishmania donovani to the sand fly vector midgut is regulated by conformational changes in the abundant surface lipophosphoglycan.. J Exp Med.

[8] Sadlova J, Price HP, Smith BA, Votypka J, Volf P (2010). The stage-regulated HASPB and SHERP proteins are essential for differentiation of the protozoan parasite Leishmania major in its sand fly vector, Phlebotomus papatasi.. Cell Microbiol.

[9] Burchmore RJ, Barrett MP (2001). Life in vacuoles–nutrient acquisition by Leishmania amastigotes.. Int J Parasitol.

[10] De Souza DP, Saunders EC, McConville MJ, Likic VA (2006). Progressive peak clustering in GC-MS Metabolomic experiments applied to Leishmania parasites.. Bioinformatics.

[11] t'Kindt R, Jankevics A, Scheltema RA, Zheng L, Watson DG (2010). Towards an unbiased metabolic profiling of protozoan parasites: optimisation of a Leishmania sampling protocol for HILIC-orbitrap analysis.. Anal Bioanal Chem.

[12] t'Kindt R, Scheltema RA, Jankevics A, Brunker K, Rijal S (2010). Metabolomics to unveil and understand phenotypic diversity between pathogen populations.. PLoS Negl Trop Dis.

[13] Saunders EC, Ng WW, Chamber JM, Ng M, Naderer T (2011). Isoptopomer profiling of Leishmania mexicana promastigotes reveals important roles for succinate fermentation and aspartate uptake in TCA cycle anaplerosis, glutamate synthesis and growth.. J Biol Chem.

[14] Naderer T, Heng J McConville MJ Evidence that intracellular stages of Leishmania major utilize amino sugars as a major carbon source.. PLoS Pathog.

[15] McConville MJ, Naderer T (2011). Metabolic Pathways Required for the Intracellular Survival of Leishmania.. Annu Rev Microbiol.

[16] Saunders EC, DP DES, Naderer T, Sernee MF, Ralton JE Central carbon metabolism of Leishmania parasites.. Parasitology.

[17] Rogers ME, Chance ML, Bates PA (2002). The role of promastigote secretory gel in the origin and transmission of the infective stage of Leishmania mexicana by the sandfly Lutzomyia longipalpis.. Parasitology.

[18] Davies CR, Cooper AM, Peacock C, Lane RP, Blackwell JM (1990). Expression of LPG and GP63 by different developmental stages of Leishmania major in the sandfly Phlebotomus papatasi.. Parasitology.

[19] Schneider P, Rosat JP, Bouvier J, Louis J, Bordier C (1992). Leishmania major: differential regulation of the surface metalloprotease in amastigote and promastigote stages.. Exp Parasitol.

[20] Pimenta PF, Saraiva EM, Sacks DL (1991). The comparative fine structure and surface glycoconjugate expression of three life stages of Leishmania major.. Exp Parasitol.

[21] Rijal S, Yardley V, Chappuis F, Decuypere S, Khanal B (2007). Antimonial treatment of visceral leishmaniasis: are current in vitro susceptibility assays adequate for prognosis of in vivo therapy outcome?. Microbes Infect.

[22] Wishart DS (2010). Computational approaches to metabolomics.. Methods Mol Biol.

[23] Zhang K, Showalter M, Revollo J, Hsu FF, Turk J (2003). Sphingolipids are essential for differentiation but not growth in Leishmania.. EMBO J.

[24] Bates PA, Rogers ME (2004). New insights into the developmental biology and transmission mechanisms of Leishmania.. Curr Mol Med.

[25] Sacks DL, Brodin TN, Turco SJ (1990). Developmental modification of the lipophosphoglycan from Leishmania major promastigotes during metacyclogenesis.. Mol Biochem Parasitol.

[26] Saraiva EM, Pimenta PF, Brodin TN, Rowton E, Modi GB (1995). Changes in lipophosphoglycan and gene expression associated with the development of Leishmania major in Phlebotomus papatasi.. Parasitology.

[27] Yao C, Donelson JE, Wilson ME (2003). The major surface protease (MSP or GP63) of Leishmania sp. Biosynthesis, regulation of expression, and function.. Mol Biochem Parasitol.

[28] Besteiro S, Williams RA, Morrison LS, Coombs GH, Mottram JC (2006). Endosome sorting and autophagy are essential for differentiation and virulence of Leishmania major.. J Biol Chem.

[29] Denny PW, Smith DF (2004). Rafts and sphingolipid biosynthesis in the kinetoplastid parasitic protozoa.. Mol Microbiol.

[30] Wassef MK, Fioretti TB, Dwyer DM (1985). Lipid analyses of isolated surface membranes of Leishmania donovani promastigotes.. Lipids.

[31] Zufferey R, Allen S, Barron T, Sullivan DR, Denny PW (2003). Ether phospholipids and glycosylinositolphospholipids are not required for amastigote virulence or for inhibition of macrophage activation by Leishmania major.. J Biol Chem.

[32] Beney L, Gervais P (2001). Influence of the fluidity of the membrane on the response of microorganisms to environmental stresses.. Appl Microbiol Biotechnol.

[33] Suutari M, Liukkonen K, Laakso S (1990). Temperature adaptation in yeasts: the role of fatty acids.. J Gen Microbiol.

[34] Turk M, Plemenitas A, Gunde-Cimerman N (2011). Extremophilic yeasts: plasma-membrane fluidity as determinant of stress tolerance.. Fungal Biol.

[35] Wahid A, Gelani S, Ashraf M, Foolad MR (2007). Heat tolerance in plants: An overview.. Environmental and Experimental Botan.

[36] Mbongo N, Loiseau PM, Billion MA, Robert-Gero M (1998). Mechanism of amphotericin B resistance in Leishmania donovani promastigotes.. Antimicrob Agents Chemother.

[37] Cauchetier E, Loiseau PM, Lehman J, Rivollet D, Fleury J (2002). Characterisation of atovaquone resistance in Leishmania infantum promastigotes.. Int J Parasitol.

[38] Rakotomanga M, Saint-Pierre-Chazalet M, Loiseau PM (2005). Alteration of fatty acid and sterol metabolism in miltefosine-resistant Leishmania donovani promastigotes and consequences for drug-membrane interactions.. Antimicrob Agents Chemother.

[39] Hannun YA, Obeid LM (2008). Principles of bioactive lipid signalling: lessons from sphingolipids.. Nat Rev Mol Cell Biol.

[40] Zhang K, Pompey JM, Hsu FF, Key P, Bandhuvula P (2007). Redirection of sphingolipid metabolism toward de novo synthesis of ethanolamine in Leishmania.. EMBO J.

[41] Zufferey R, Mamoun CB (2002). Choline transport in Leishmania major promastigotes and its inhibition by choline and phosphocholine analogs.. Mol Biochem Parasitol.

[42] Rifkin MR, Fairlamb AH (1985). Transport of ethanolamine and its incorporation into the variant surface glycoprotein of bloodstream forms of Trypanosoma brucei.. Mol Biochem Parasitol.

[43] Zhang K, Hsu FF, Scott DA, Docampo R, Turk J (2005). Leishmania salvage and remodelling of host sphingolipids in amastigote survival and acidocalcisome biogenesis.. Mol Microbiol.

[44] Gibellini F, Smith TK The Kennedy pathway–De novo synthesis of phosphatidylethanolamine and phosphatidylcholine.. IUBMB Life.

[45] Saint-Pierre-Chazalet M, Ben Brahim M, Le Moyec L, Bories C, Rakotomanga M (2009). Membrane sterol depletion impairs miltefosine action in wild-type and miltefosine-resistant Leishmania donovani promastigotes.. J Antimicrob Chemother.

[46] Opperdoes FR, Coombs GH (2007). Metabolism of Leishmania: proven and predicted.. Trends Parasitol.

[47] Saunders EC, Ng WW, Chamber JM, Ng M, Naderer T Isoptopomer profiling of Leishmania mexicana promastigotes reveals important roles for succinate fermentation and aspartate uptake in TCA cycle anaplerosis, glutamate synthesis and growth.. J Biol Chem.

[48] Bonafe L, Troxler H, Kuster T, Heizmann CW, Chamoles NA (2000). Evaluation of urinary acylglycines by electrospray tandem mass spectrometry in mitochondrial energy metabolism defects and organic acidurias.. Mol Genet Metab.

[49] Sengupta A (2010). A Urine IH NMR based Metabonomic Approach to Understand the Host Metabolic Response towards Plasmodium vivax Infection.. Proceedings of 2010 International Conference on Systems in Medicine and Biology.

[50] Cunningham ML, Beverley SM (2001). Pteridine salvage throughout the Leishmania infectious cycle: implications for antifolate chemotherapy.. Mol Biochem Parasitol.

[51] Cunningham ML, Titus RG, Turco SJ, Beverley SM (2001). Regulation of differentiation to the infective stage of the protozoan parasite Leishmania major by tetrahydrobiopterin.. Science.

[52] Landfear SM, Ullman B, Carter NS, Sanchez MA (2004). Nucleoside and nucleobase transporters in parasitic protozoa.. Eukaryot Cell.

[53] Ortiz D, Sanchez MA, Pierce S, Herrmann T, Kimblin N (2007). Molecular genetic analysis of purine nucleobase transport in Leishmania major.. Mol Microbiol.

[54] Naderer T, McConville MJ (2008). The Leishmania-macrophage interaction: a metabolic perspective.. Cell Microbiol.

[55] McConville MJ, de Souza D, Saunders E, Likic VA, Naderer T (2007). Living in a phagolysosome; metabolism of Leishmania amastigotes.. Trends Parasitol.

[56] Rosenzweig D, Smith D, Opperdoes F, Stern S, Olafson RW (2008). Retooling Leishmania metabolism: from sand fly gut to human macrophage.. FASEB J.

[57] Zhang K, Beverley SM (2010). Phospholipid and sphingolipid metabolism in Leishmania.. Mol Biochem Parasitol.

[58] Colotti G, Ilari A (2011). Polyamine metabolism in Leishmania: from arginine to trypanothione.. Amino Acids.

