# Supplementary figures and images for: Metabolic Variation during Development in Culture of Leishmania donovani Promastigotes
Source: PLoS Negl Trop Dis. 2011 Dec 20;5(12):e1451. doi: 10.1371/journal.pntd.0001451 (PMC3243725; doi:10.1371/journal.pntd.0001451)

Figure S1

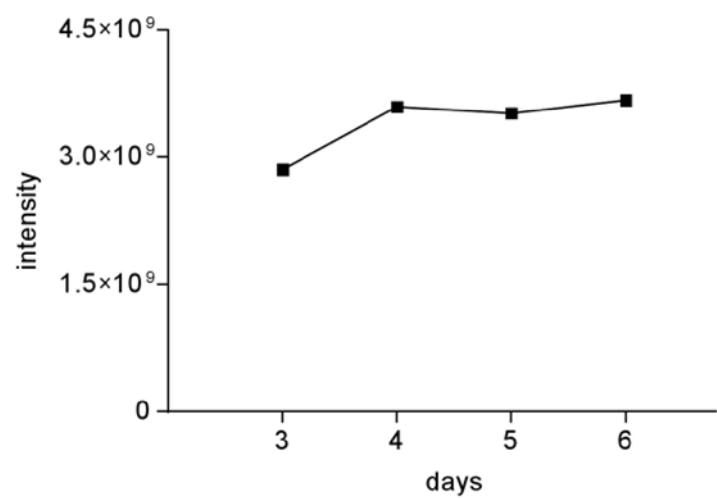

Supplement: Figure S1 — Overall metabolite levels remain similar during L. donovani promastigotes development in vitro . Sum of total metabolite intensities from L. donovani promastigotes identified by LC-MS analysis at days 3, 4, 5 or 6 of in vitro growth. (PDF) [file pntd.0001451.s001.pdf]

**Figure S2**

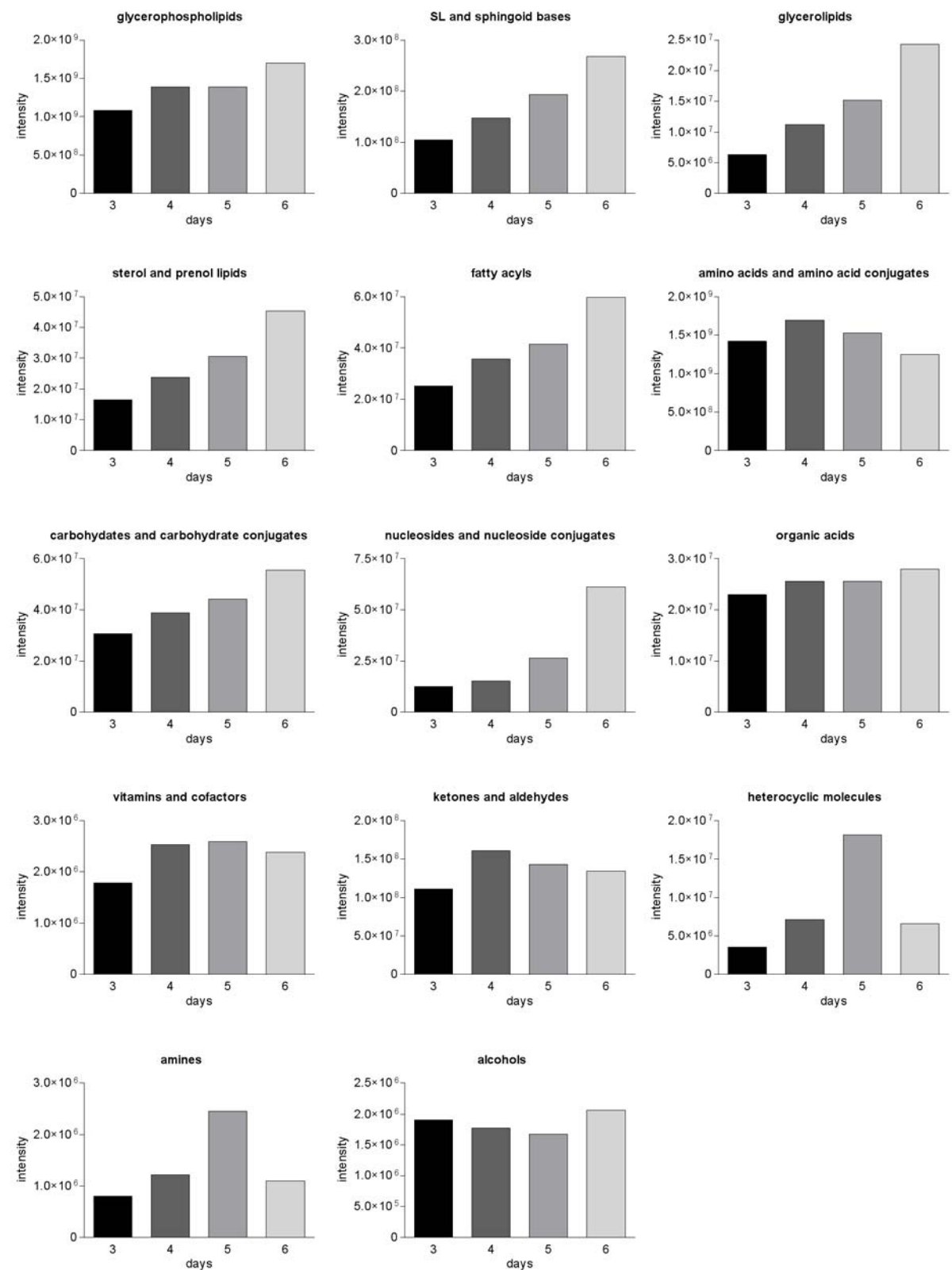

Supplement: Figure S2 — Overall metabolite levels for each compound category during L. donovani promastigotes development in vitro . Sum of total metabolite intensities from L. donovani promastigotes identified by LC-MS analysis at days 3, 4, 5 or 6 of in vitro growth grouped by compound categories. (PDF) [file pntd.0001451.s002.pdf]

Figure S3

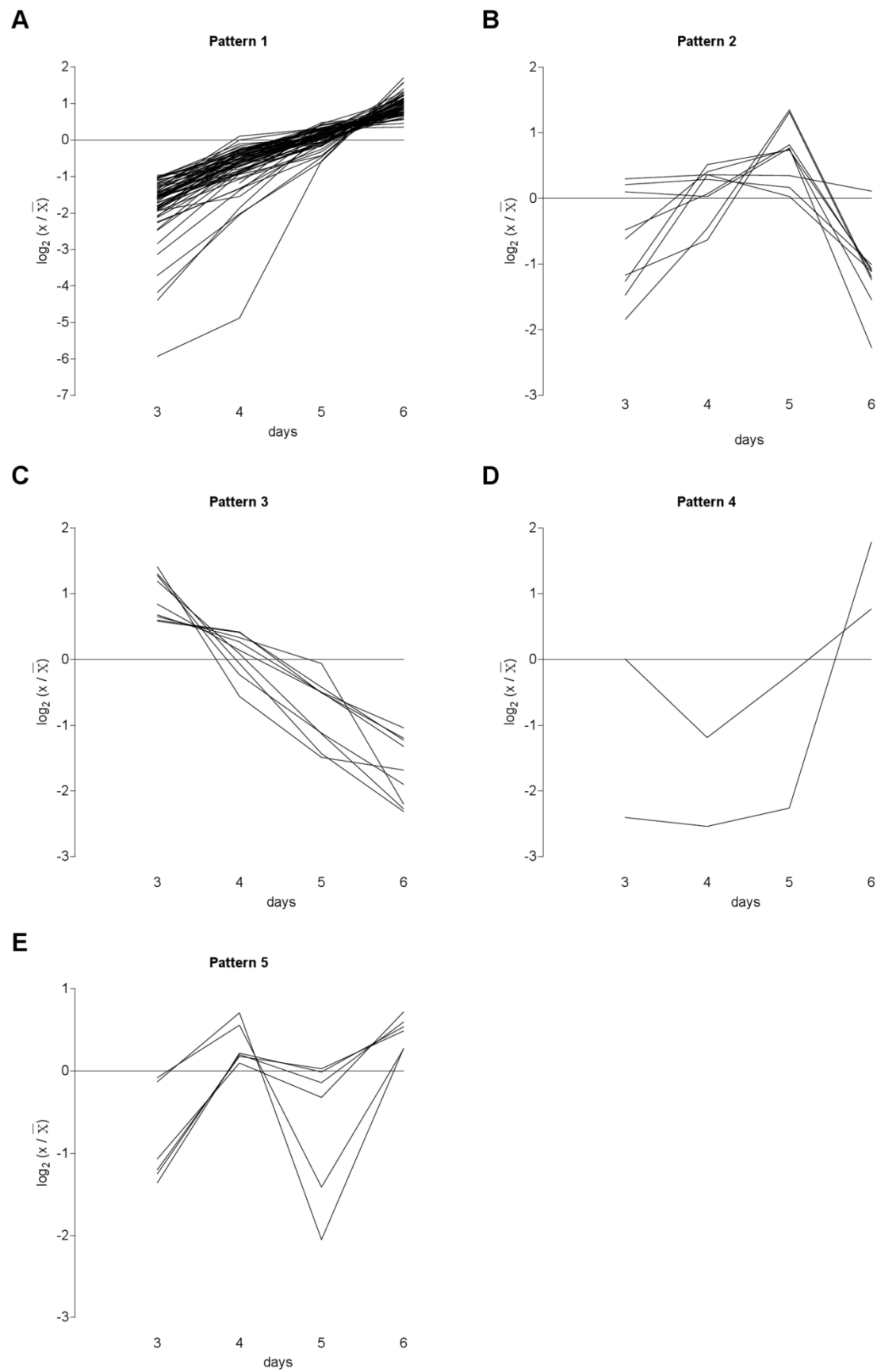

Supplement: Figure S3 — Patterns of metabolite variation in level during L. donovani promastigote in vitro growth from day 3 to day 6. Analysis of L. donovani promastigotes metabolome at day 3, 4, 5 and 6 allowed the distinction of 5 patterns (1–5) based on the metabolic profile for each metabolite that differed in intensity by 2-fold or more from the mean levels in at least one of the time points analyzed (a total of 97 metabolites). The relative level of each metabolite is shown, following logarithmic transformation (base 2); log2 (x/ 3–6) below −1 or above 1 indicates a 2-fold change; x, time point analyzed (day 3, 4, 5 or 6). (A) Pattern 1 (74% of all metabolites considered) and (C) pattern 3 (9%) include metabolites that increased or decreased, respectively, continuously during all the whole period analyzed; (B) pattern 2 (9%) include metabolites which had peak levels on days 4 or 5 but which then declined; (D) pattern 4 (2%) metabolite levels decreased from day 3 to day 4 and then increased and (E) pattern 5 (6%) include metabolites that showed an increase followed by a decrease and then another increase. (PDF) [file pntd.0001451.s003.pdf]

### Figure S4

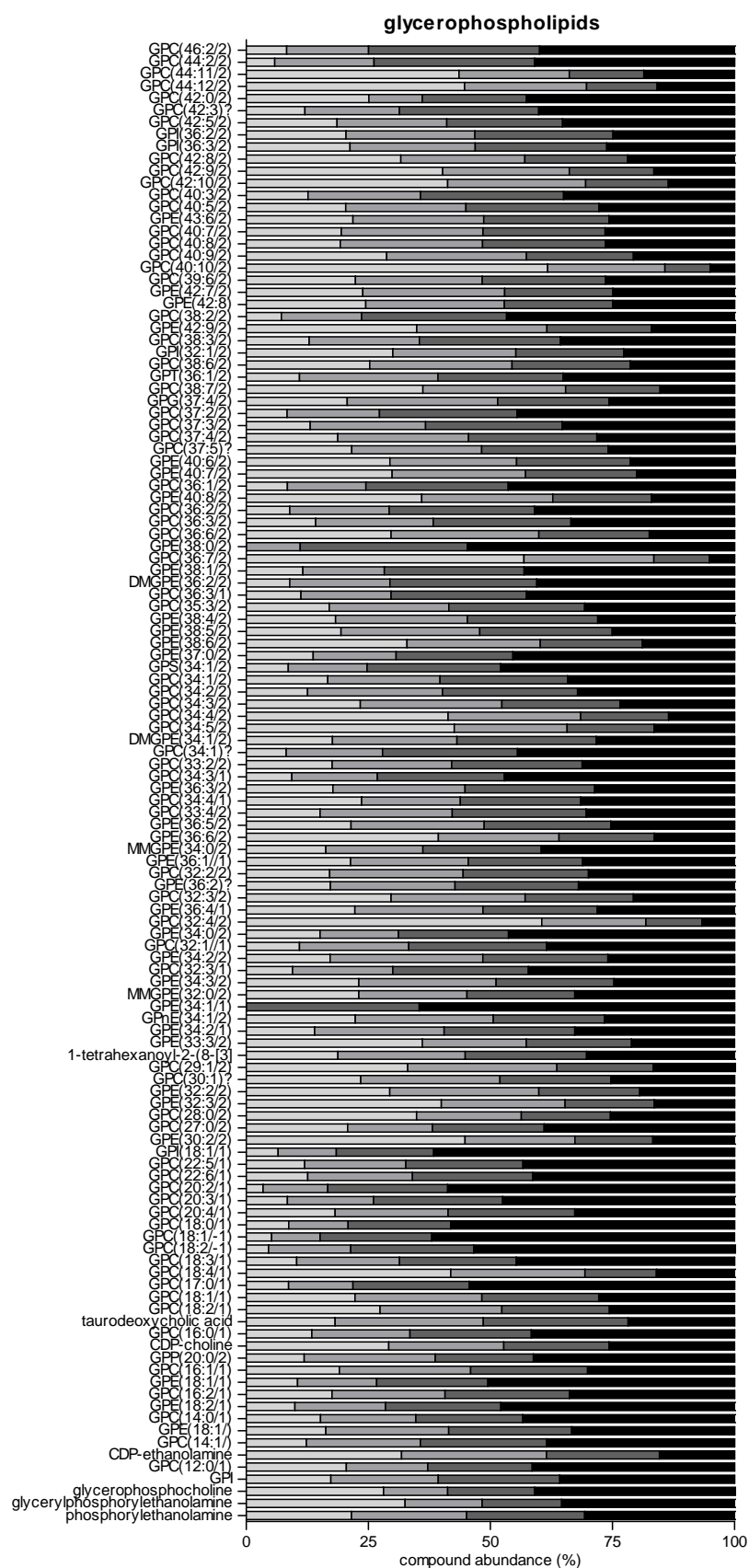

Supplement: Figure S4 — Glycerophospholipids abundance during L. donovani promastigotes development in vitro . Glycerophospholipids abundance is represented for each metabolite on each day as a percentage of the total abundance detected, which comprises the sum of the total metabolite intensity during the 4-day period of analysis. (PDF) [file pntd.0001451.s004.pdf]

**Figure S5**

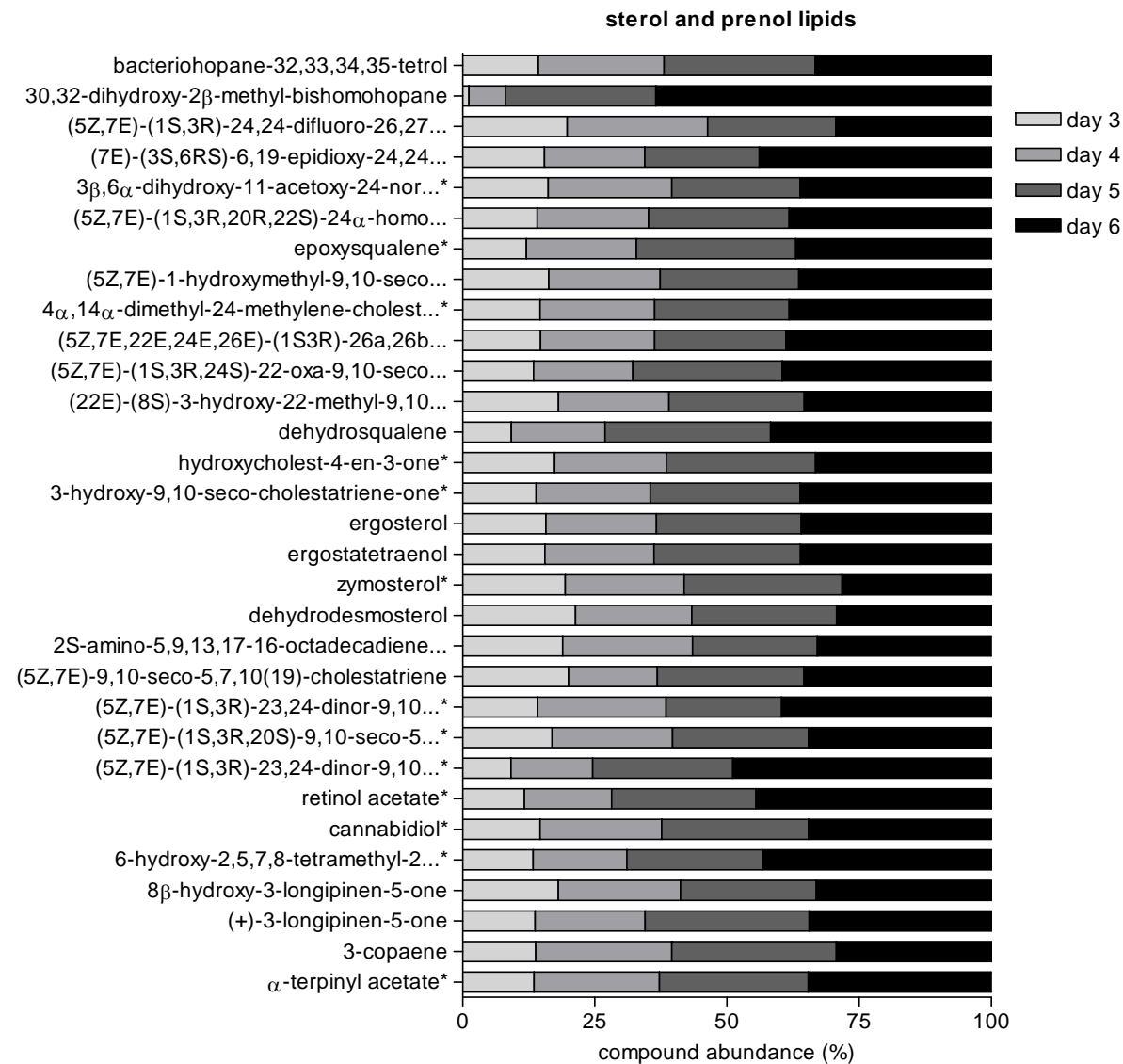

Supplement: Figure S5 — Sterol and prenol lipids abundance during L. donovani promastigotes development in vitro . Sterol and prenol lipids abundance is represented for each metabolite on each day as a percentage of the total abundance detected, which comprises the sum of the total metabolite intensity during the 4-day period of analysis. Metabolites labelled with * represent peaks with multiple potential identifications, for which just one is shown in this figure but the full list is given in Tables S1 and S2. (PDF) [file pntd.0001451.s005.pdf]

**Figure S6**

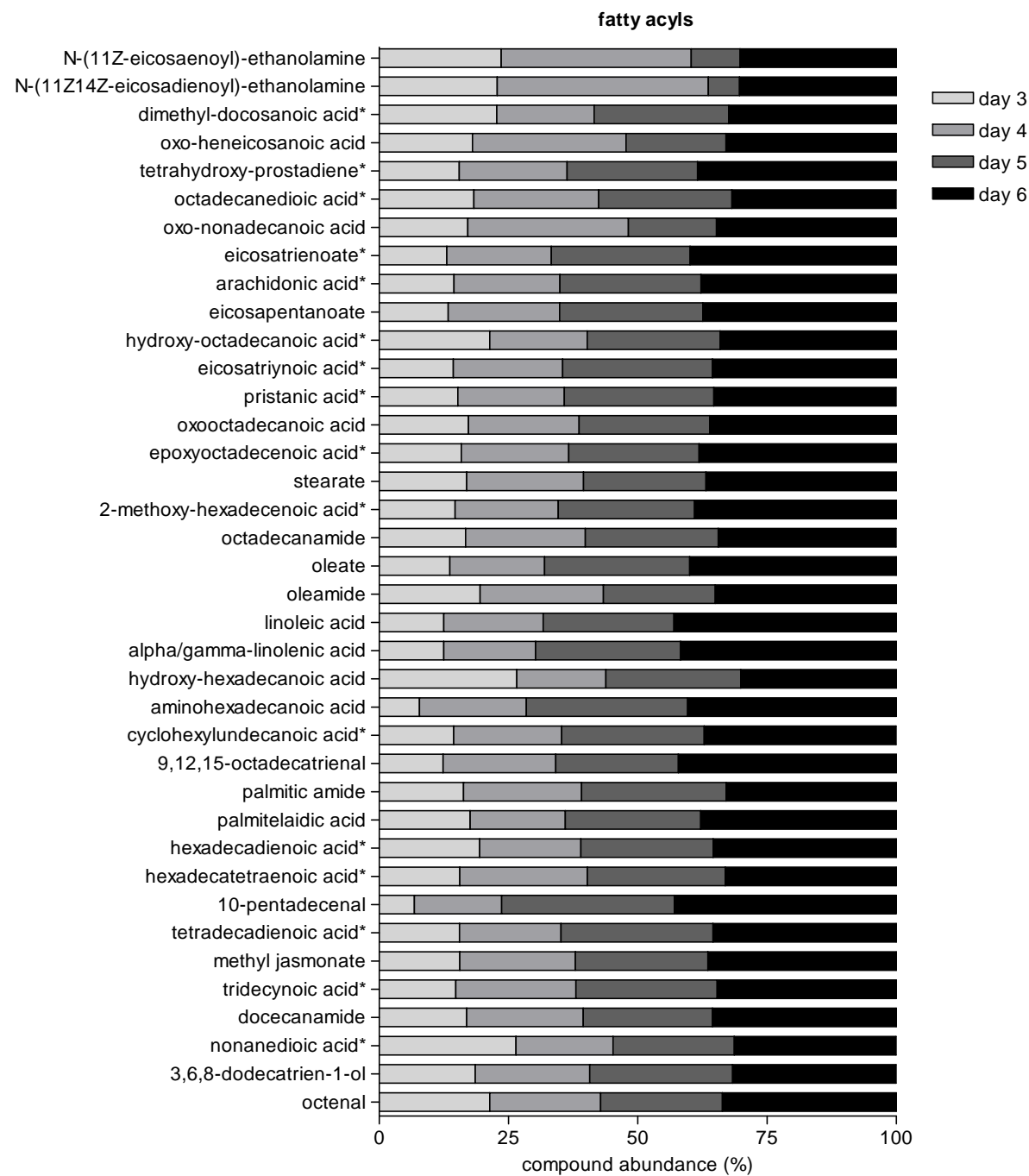

Supplement: Figure S6 — Fatty acyls abundance during L. donovani promastigotes development in vitro . Fatty acyls abundance is represented for each metabolite on each day as a percentage of the total abundance detected, which comprises the sum of the total metabolite intensity during the 4-day period of analysis. Metabolites labelled with * represent peaks with multiple potential identifications, for which just one is shown in this figure but the full list is given in Tables S1 and S2. (PDF) [file pntd.0001451.s006.pdf]

**Figure S7**

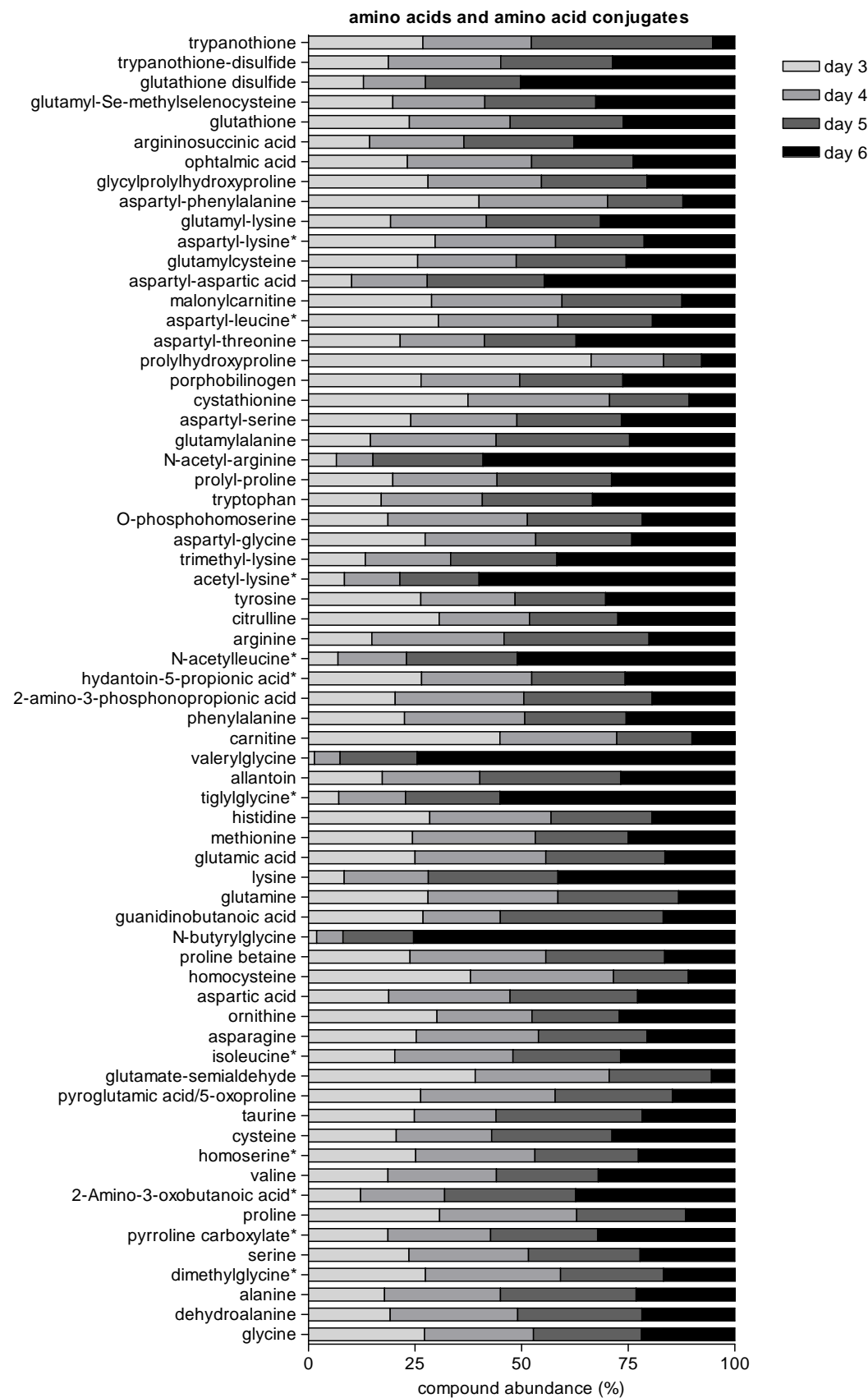

Supplement: Figure S7 — Amino acids and amino acids conjugates abundance during L. donovani promastigotes development in vitro . Amino acids and amino acids conjugates abundance is represented for each metabolite on each day as a percentage of the total abundance detected, which comprises the sum of the total metabolite intensity during the 4-day period of analysis. Metabolites labelled with * represent peaks with multiple potential identifications, for which just one is shown in this figure but the full list is given in Tables S1 and S2. (PDF) [file pntd.0001451.s007.pdf]

**Figure S8**

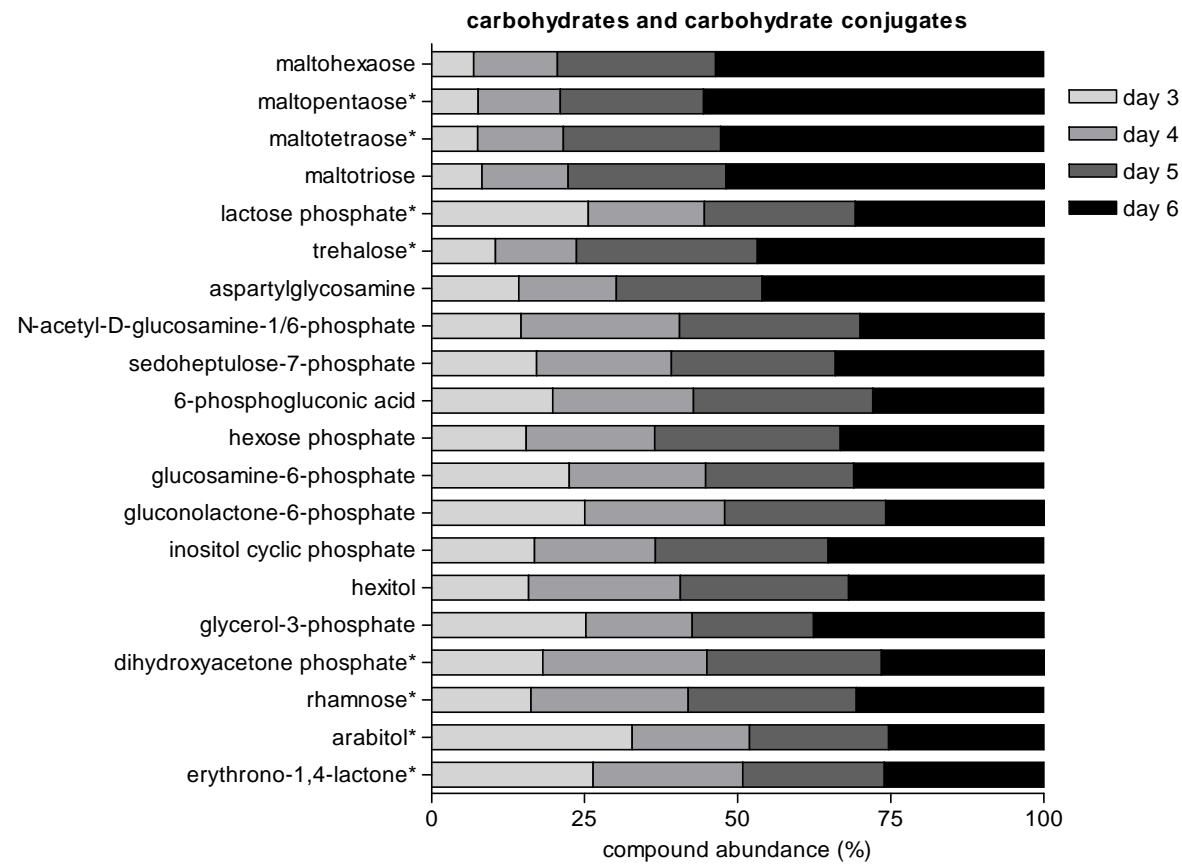

Supplement: Figure S8 — Carbohydrates and carbohydrate conjugates abundance during L. donovani promastigotes development in vitro . Carbohydrates and carbohydrate conjugates abundance is represented for each metabolite on each day as a percentage of the total abundance detected, which comprises the sum of the total metabolite intensity during the 4-day period of analysis. Metabolites labelled with * represent peaks with multiple potential identifications, for which just one is shown in this figure but the full list is given in Tables S1 and S2. (PDF) [file pntd.0001451.s008.pdf]

**Figure S9**

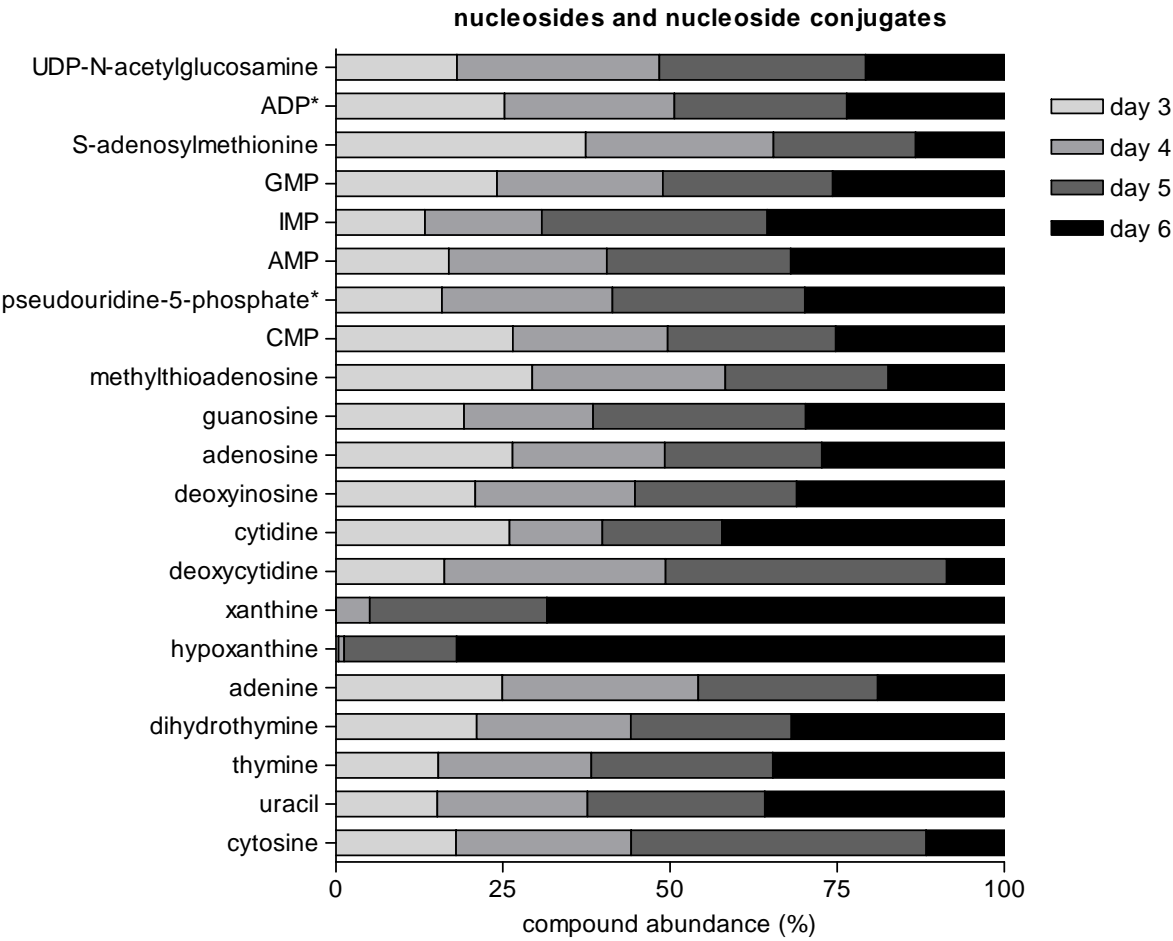

Supplement: Figure S9 — Nucleosides and nucleoside conjugates abundance during L. donovani promastigotes development in vitro . Nucleosides and nucleoside conjugate metabolites abundance is represented for each metabolite on each day as a percentage of the total abundance detected, which comprises the sum of the total metabolite intensity during the 4-day period of analysis. Metabolites labelled with * represent peaks with multiple potential identifications, for which just one is shown in this figure but the full list is given in Tables S1 and S2. (PDF) [file pntd.0001451.s009.pdf]

Figure S10

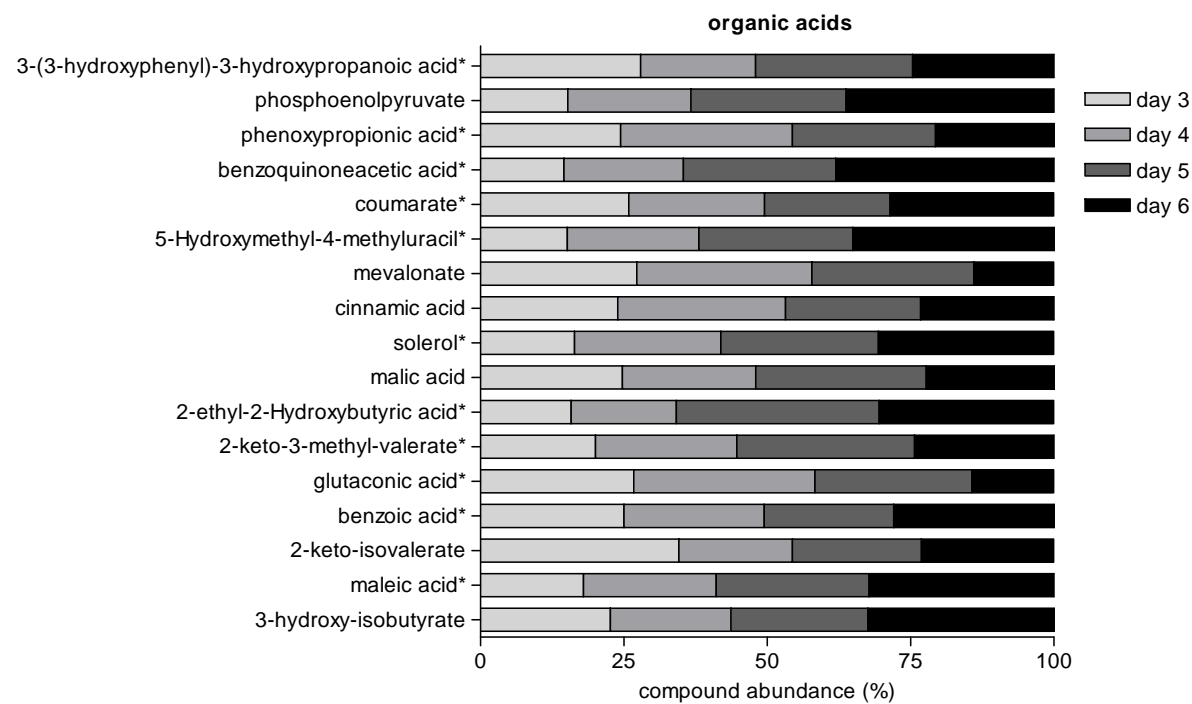

Supplement: Figure S10 — Organic acids abundance during L. donovani promastigotes development in vitro . Organic acids abundance is represented for each metabolite on each day as a percentage of the total abundance detected, which comprises the sum of the total metabolite intensity during the 4-day period of analysis. Metabolites labelled with * represent peaks with multiple potential identifications, for which just one is shown in this figure but the full list is given in Tables S1 and S2. (PDF) [file pntd.0001451.s010.pdf]

Figure S11

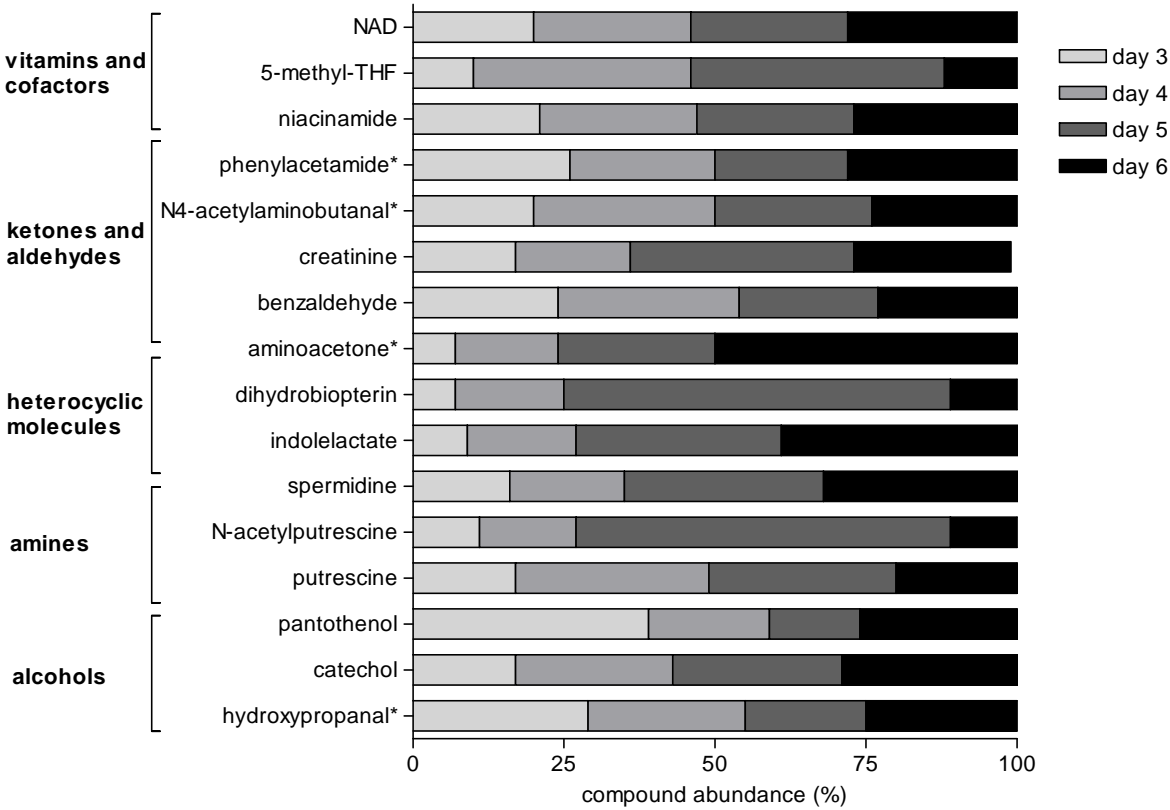

Supplement: Figure S11 — Vitamins and cofactors, ketones and aldehydes, heterocyclic molecules, amines and alcohols abundance during L. donovani promastigotes development in vitro . Vitamins and cofactors, ketones and aldehydes, heterocyclic molecules, amines and alcohols abundance is represented for each metabolite on each day as a percentage of the total abundance detected, which comprises the sum of the total metabolite intensity during the 4-day period of analysis. Metabolites labelled with * represent peaks with multiple potential identifications, for which just one is shown in this figure but the full list is given in Tables S1 and S2. (PDF) [file pntd.0001451.s011.pdf]
