# Supplementary material for: Metabolic Variation during Development in Culture of Leishmania donovani Promastigotes
Source: PLoS Negl Trop Dis. 2011 Dec 20;5(12):e1451. doi: 10.1371/journal.pntd.0001451 (PMC3243725; doi:10.1371/journal.pntd.0001451)
Supplement: Table S3 — Intensity levels of metabolites that differ significantly during L. donovani promastigote development in vitro . Metabolites are ordered by compound category; for each metabolite is shown the average intensity per 25 µg cell protein at days 3, 4, 5 and 6 and the respective metabolic profile pattern (analysis shown in Figure S2). Metabolites labelled with * represent peaks with multiple potential identifications, for which just one is shown in this figure but the full list is given in Table S1 and S2. (PDF) [file pntd.0001451.s014.pdf]

Table S3

	Average intensity per 25µg cell protein				
	day 3	day 4	day 5	day 6	pattern
**amines**					
N-acetylputrescine	2.53E+05	3.69E+05	1.42E+06	2.42E+05	2
**amino acids and amino acids conjugates**					
proline	2.95E+08	3.07E+08	2.45E+08	1.10E+08	2
2-amino-3-oxobutanoic acid*	2.29E+05	3.69E+05	5.77E+05	6.99E+05	1
glutamate-semialdehyde	1.36E+07	1.09E+07	8.36E+06	1.89E+06	3
homocysteine	2.56E+05	2.26E+05	1.18E+05	7.34E+04	3
N-butyrylglycine	1.28E+05	4.13E+05	1.11E+06	5.04E+06	1
lysine	5.54E+06	1.31E+07	2.01E+07	2.75E+07	1
tiglylglycine*	7.76E+04	1.70E+05	2.41E+05	6.00E+05	1
valerylglycine	1.64E+05	7.21E+05	2.16E+06	8.90E+06	1
carnitine	1.89E+06	1.16E+06	7.44E+05	4.24E+05	3
N-acetylleucine*	8.48E+04	1.97E+05	3.19E+05	6.26E+05	1
acetyl-lysine*	1.37E+05	2.11E+05	3.01E+05	9.75E+05	1
N-acetyl-arginine	7.16E+04	9.36E+04	2.82E+05	6.45E+05	1
cystathionine	7.54E+07	6.69E+07	3.76E+07	2.15E+07	3
prolylhydroxyproline	3.64E+06	9.33E+05	4.89E+05	4.28E+05	3
malonylcarnitine	3.10E+05	3.29E+05	3.02E+05	1.33E+05	2
aspartyl-aspartic acid	2.44E+05	4.27E+05	6.60E+05	1.08E+06	1
aspartyl-phenylalanine	1.20E+07	9.04E+06	5.29E+06	3.64E+06	3
glutathione disulfide	4.47E+04	5.03E+04	7.74E+04	1.74E+05	1
trypanothione	1.32E+06	1.25E+06	2.09E+06	2.55E+05	2
**carbohydrates and carbohydrate conjugates**					
trehalose*	4.18E+04	5.29E+04	1.18E+05	1.87E+05	1
maltotriose	1.30E+05	2.21E+05	4.06E+05	8.18E+05	1
maltotetraose*	2.15E+05	3.97E+05	7.31E+05	1.50E+06	1
maltopentaose*	5.27E+05	9.24E+05	1.61E+06	3.84E+06	1
maltohexaose	7.40E+04	1.46E+05	2.78E+05	5.75E+05	1
**fatty acyls**					
10-pentadecenal	8.05E+05	1.99E+06	3.95E+06	5.07E+06	1
9, 12, 15-octadecatrienal*	4.12E+05	7.24E+05	7.89E+05	1.40E+06	1
aminohexadecanoic acid	1.24E+06	3.28E+06	4.95E+06	6.42E+06	1
alpha/gamma-linolenic acid	3.69E+05	5.26E+05	8.29E+05	1.23E+06	1
linoleic acid	4.13E+05	6.35E+05	8.33E+05	1.42E+06	1
N-(11Z, 14Z-eicosadienoyl)-ethanolamine	1.25E+06	2.24E+06	3.32E+05	1.66E+06	5
N-(11Z-eicosaenoyl)-ethanolamine	4.26E+05	6.65E+05	1.71E+05	5.46E+05	5
**glycerolipids**					
DAG(36:5)	2.75E+05	6.27E+05	9.69E+05	1.49E+06	1
DAG(36:4)	2.41E+05	6.16E+05	1.10E+06	2.08E+06	1
DAG(36:3)	2.01E+05	4.02E+05	6.79E+05	1.24E+06	1
DAG(42:3)*	2.07E+05	1.87E+05	2.28E+05	3.74E+06	4
DAG(46:7)	1.18E+06	3.49E+06	5.64E+06	7.39E+06	1
TAG(54:7)	9.96E+04	1.76E+05	2.99E+05	5.63E+05	1
TAG(54:6)	9.75E+04	2.42E+05	2.75E+05	5.33E+05	1
TAG(54:5)	1.05E+05	1.34E+05	2.41E+05	4.09E+05	1

	Average intensity per 25μg cell protein				
	**day 3**	**day 4**	**day 5**	**day 6**	**pattern**
**glycerophospholipids**					
GPC(14:1)	1.63E+05	3.07E+05	3.41E+05	5.07E+05	1
lysoPE(18:2/1)	5.44E+05	1.00E+06	1.29E+06	2.60E+06	1
GPE(18:1/1)	4.08E+05	6.25E+05	8.82E+05	1.96E+06	1
GPP(20:0/2)	1.08E+05	2.45E+05	1.83E+05	3.76E+05	5
GPC(17:0/1)	1.35E+05	2.05E+05	3.70E+05	8.45E+05	1
GPC(18:3/1)	1.80E+06	3.66E+06	4.16E+06	7.79E+06	1
GPC(18:2/-1)	2.57E+06	9.45E+06	1.41E+07	3.00E+07	1
GPC(18:1/-1)	1.03E+06	1.96E+06	4.49E+06	1.22E+07	1
GPC(18:0/1)	2.37E+06	3.32E+06	5.71E+06	1.58E+07	1
lysoPC(20:3/1)	4.60E+05	9.64E+05	1.44E+06	2.59E+06	1
lysoPC (20:2/1)	2.17E+05	8.20E+05	1.52E+06	3.65E+06	1
GPC(22:5/1)	1.60E+06	2.76E+06	3.20E+06	5.78E+06	1
GPI(18:1/1)	7.03E+05	1.28E+06	2.15E+06	6.64E+06	1
GPC(32:3/1)	5.42E+06	1.17E+07	1.58E+07	2.41E+07	1
GPC(32:1//1)*	7.21E+06	1.48E+07	1.86E+07	2.55E+07	1
GPC(32:4/2)*	7.73E+06	2.72E+06	1.47E+06	8.56E+05	3
GPC(34:3/1)*	1.94E+06	3.61E+06	5.38E+06	9.75E+06	1
GPC(34:1)*	1.81E+06	4.36E+06	6.10E+06	9.81E+06	1
GPS(34:1/2)	4.93E+04	9.19E+04	1.56E+05	2.73E+05	1
GPC(36:3/1)	8.31E+05	1.36E+06	2.05E+06	3.16E+06	1
DMGPE(36:2/2)*	3.04E+06	7.00E+06	1.02E+07	1.38E+07	1
GPE(38:1/2)*	7.82E+05	1.12E+06	1.93E+06	2.91E+06	1
GPC(36:7/2)	1.77E+07	8.26E+06	3.54E+06	1.61E+06	3
GPC(36:2/2)	3.92E+07	8.97E+07	1.31E+08	1.80E+08	1
GPC(36:1/2)	7.42E+06	1.42E+07	2.57E+07	4.08E+07	1
GPC(37:2/2)*	4.74E+05	1.07E+06	1.59E+06	2.52E+06	1
GPT(36:1/2)	1.11E+06	2.89E+06	2.61E+06	3.58E+06	5
GPC(38:2/2)	1.46E+06	3.34E+06	6.01E+06	9.48E+06	1
GPC(40:10/2)	1.61E+07	6.25E+06	2.41E+06	1.31E+06	3
GPC(42:3)	2.29E+05	3.68E+05	5.41E+05	7.63E+05	1
GPC(42:0/2)	1.61E+05	7.04E+04	1.37E+05	2.74E+05	4
GPC(44:2/2)	3.37E+05	1.17E+06	1.90E+06	2.36E+06	1
GPC(46:2/2)	8.51E+04	1.72E+05	3.58E+05	4.09E+05	1
**heterocyclic molecules**					
indolelactate	2.40E+05	4.93E+05	9.25E+05	1.08E+06	1
dihydrobiopterin	1.61E+06	4.21E+06	1.46E+07	2.49E+06	2
**ketones and aldehydes**					
aminoacetone*	6.33E+05	1.66E+06	2.54E+06	4.79E+06	1
**nucleosides and nucleoside conjugates**					
cytosine	4.23E+05	6.16E+05	1.04E+06	2.74E+05	2
hypoxanthine	1.97E+05	4.06E+05	8.04E+06	3.92E+07	1
deoxycytidine	2.58E+05	5.26E+05	6.69E+05	1.37E+05	2
**others**					
diphosphate	2.29E+05	8.11E+05	8.09E+05	6.86E+05	2
N-methylpelletierine	6.41E+04	1.19E+05	2.23E+05	8.01E+05	1

	Average intensity per 25µg cell protein				
	**day 3**	**day 4**	**day 5**	**day 6**	**pattern**
**sphingolipids and sphingoid bases**					
1-deoxy-sphinganine	7.66E+05	1.78E+06	2.56E+06	4.22E+06	1
heptadecasphinganine	2.03E+05	3.77E+05	6.85E+05	1.59E+06	1
dehydrosphinganine	5.47E+05	1.62E+06	2.57E+06	3.41E+06	1
SP(2-amino-14, 16-dimethyloctadecan-3-ol)	1.97E+05	3.43E+05	5.10E+05	7.92E+05	1
4-hydroxysphinganine	1.54E+05	4.09E+05	5.08E+05	5.61E+05	1
N, N-dimethylsphing-4-enine	1.66E+05	4.56E+05	3.60E+05	6.02E+05	5
SP(2S-dimethylaminooctadecane-1, 3R-diol)	9.15E+05	1.77E+06	2.59E+06	3.07E+06	1
N-(hexadecanoyl)-sphing-4-enine	4.60E+06	1.08E+07	2.28E+07	4.17E+07	1
N-(hexadecanoyl)-4S-hydroxysphinganine	1.99E+06	6.10E+06	1.36E+07	2.24E+07	1
N-(octadecanoyl)-sphing-4-enine	1.41E+05	4.74E+05	1.46E+06	2.87E+06	1
**sterol and prenol lipids**					
retinol acetate*	3.05E+05	4.31E+05	7.13E+05	1.16E+06	1
(5Z, 7E)-(1S, 3R)-23, 24-dinor-9, 10-seco…*	2.00E+05	3.37E+05	5.79E+05	1.07E+06	1
dehydrosqualene	4.84E+05	9.31E+05	1.65E+06	2.19E+06	1
epoxysqualene*	2.21E+05	3.80E+05	5.52E+05	6.77E+05	1
30,32-dihydroxy-2b-methyl-bishomohopane	1.40E+05	8.12E+05	3.36E+06	7.48E+06	1
**vitamins and cofactors**					
5-methyl-THF	1.31E+05	4.47E+05	5.24E+05	1.49E+05	2
